# Fibrosis-memory is mediated by IL-3–producing T cells and drives progression of fibrosis

**DOI:** 10.1172/JCI192095

**Published:** 2026-03-16

**Authors:** Simone Buchtler, Antje Frühauf, Sophia Neumayer, Kathrin Schmidbauer, Yvonne Talke, Frederike Winter-Köhler, Saidou Balam, Karin Landgraf, Claudia Gebhard, Michael Rehli, Florian Volker Schlieckau, Maria Beck, Florian Günther, Martin Fleck, Kerstin Renner, Matthias Mack

**Affiliations:** 1 Department of Nephrology, University Hospital Regensburg, Regensburg, Germany.; 2 LIT Leibnitz Institute of Immunotherapy, Regensburg, Germany.; 3 Department of Internal Medicine III, University Hospital Regensburg, Regensburg, Germany.; 4 Department of Rheumatology/Clinical Immunology, Asklepios Clinic, Bad Abbach, Germany.; 5 Department of Internal Medicine I, University Hospital Regensburg, Regensburg, Germany.

**Keywords:** Immunology, Nephrology, Fibrosis

## Abstract

Repetitive injuries are an important trigger of progressive fibrosis. To study if repetitive injuries induce an accelerated profibrotic process, also called “fibrosis-memory,” we established an experimental system with two consecutive, clearly separated insults in a model of renal fibrosis with reversible and irreversible unilateral ureteral obstruction. We found that a preceding fibrotic event of one kidney markedly enhanced subsequent development of fibrosis in the contralateral kidney. Aggravation of fibrosis during the second insult was dependent on memory CD4^+^ T cells. T cell depletion abrogated the fibrosis-memory effect, while adoptive transfer of memory T cells from fibrotic mice enhanced fibrosis in the recipients. Moreover, IL-3 production by memory CD4^+^ T cells was essential for aggravation of fibrosis in memory situations. In patients with systemic sclerosis, IL-3 expression by T cells was markedly increased, especially after a long disease duration accompanied by involvement of internal organs. In summary, our data identify IL-3–mediated fibrosis-memory as an important driver of progressive fibrosis.

## Abstract 2

Following renal damage mice develop a fibrosis-memory aggravating fibrosis during a second damage via IL-3 producing memory CD4+ T cells

## Introduction

Organ fibrosis is observed in various chronic diseases and is considered as a leading cause of organ failure and morbidity (1, 2). While a single and short injury rarely results in relevant fibrosis, prolonged or repetitive injuries are important drivers of organ fibrosis (3–5). Repetitive injuries add up not only in a linear manner, but initiate an accelerated and enhanced profibrotic process (6, 7). So far, the underlying mechanisms of organ fibrosis have been studied primarily at the level of fibroblasts. It has been shown that fibroblasts undergo epigenetic and metabolic changes in response to profibrotic signals that result in the production of more extracellular matrix in vitro (8–11). Such a “priming” of fibroblasts, also called “fibroblast-memory,” is considered an attractive concept to aid in the understanding of chronic fibrotic diseases. However, strong evidence for its relevance in vivo is lacking, and it is also difficult to imagine how changes in resident fibroblasts could lead to spreading of fibrosis across whole organs or even multiple organ systems, as seen for instance in patients with systemic sclerosis (SSc).

We therefore established an experimental system to investigate whether fibrosis-memory occurs in vivo and, if so, to understand the underlying mechanisms. We induced two defined fibrotic injuries and asked whether the second injury would result in more fibrosis than the first one. We also aimed to avoid colocalization of the second injury with the first one, as it would be difficult to quantify incremental fibrosis on top of a preexisting fibrosis. Therefore, we did or did not induce a reversible unilateral ureteral obstruction (RUUO) to one kidney (first fibrotic insult) (12) and after various periods of time induced an unilateral ureteral obstruction (UUO) to the contralateral kidney (second fibrotic insult). We then analyzed whether a first fibrotic insult aggravated development of fibrosis during the second insult. Thus, the model revealed the affect of recurrent fibrotic insults on development of fibrosis in the same organ system (kidney). UUO leads to damage of tubular epithelial cells, rapid influx of leukocytes, and development of a highly reproducible fibrosis of the affected kidney within 6–7 days (13). Recurrent episodes of kidney injury are known to markedly increase the risk for renal failure (14). Each episode of kidney injury doubles the risk of developing stage 4 renal failure (15), and short intervals between two episodes are more harmful than longer ones (16).

We show that a preceding fibrotic event enhanced development of fibrosis in the contralateral kidney during a subsequent UUO injury. This memory effect persisted for several weeks and was associated with a more pronounced infiltration of T cells and monocytes into the damaged kidney. Depletion of T cells but not monocytes abrogated fibrosis-memory, while adoptive transfer of memory T cells from fibrotic mice enhanced fibrosis. The profibrotic capacity of CD4^+^ T cells was almost completely dependent on their release of IL-3, since no aggravation of fibrosis occurred in the absence of IL-3. In patients with SSc, T cell–derived expression of IL-3 was markedly increased, especially after prolonged disease duration and with more widespread organ involvement, suggesting that fibrosis-memory was generated over time. Overall, our data show that T cell–derived IL-3 may be an attractive target to interfere with fibrosis-memory and progression of fibrosis.

## Results

### Development of fibrosis is enhanced by a preceding fibrotic event.

**Figure 1:**
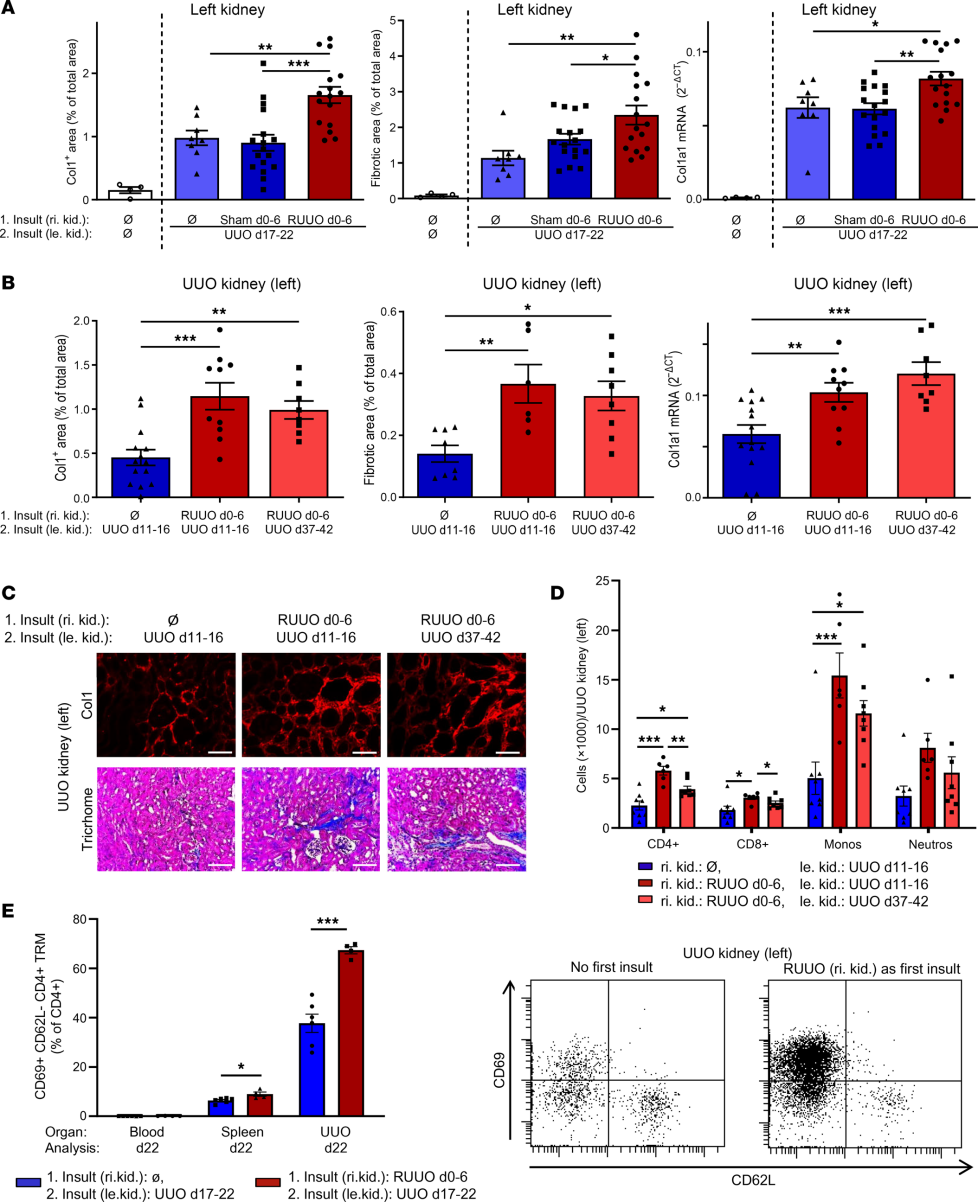
Evidence for long-lasting fibrosis-memory in mice with renal fibrosis. (**A**, **B**, and **E**) Mice underwent RUUO of the right kidney from day 0–6 (first insult), were sham operated, or remained naive (Ø). Mice then underwent UUO of the left kidney from day 17 to 22 (second insult) or remained naive (Ø). (**A**) Quantification of collagen-1 (Col1), overall fibrosis (fibrotic area), and col1a1 mRNA expression in the left kidney on day 22. Two pooled experiments, with *n* = 4, naive; 8, received the second insult only; 17, sham; and 16, RUUO. (**B**–**D**) Mice underwent RUUO of the right kidney from day 0 to 6 (first insult) or remained naive (Ø). Mice then underwent UUO of the left kidney from day 11 to 16 or from day 37 to 42 (second insult). (**B**) Analysis of the left UUO kidney on day 16 or day 42. Two pooled experiments, with *n* = 14 naive; 10 day 16 UUO; and 8 day 37 UUO for immunofluorescence and qPCR, and 1 single experiment for Trichrome staining, with *n* = 8 received the second insult only; 6, day 11 UUO; and 8, day 37 UUO. (**C**) Representative images of collagen-1 immunofluorescence and Trichrome staining. (**D**) Quantification of infiltrating CD4^+^ T cells, CD8^+^ T cells, monocytes (Monos), and neutrophils (Neutros) in the left UUO kidneys (single experiment with *n* = 8 received the second insult only; 6, day 11 UUO; and 8, day 37 UUO). (**E**) Quantification of CD4^+^ tissue-resident memory (TRM) T cells in the blood, spleen, and left UUO kidneys (single experiment with *n* = 6 and 4). Scale bars: 100 μm. Data are represented as mean ± SEM. One-way ANOVA with multiple comparisons. *P < 0.05; **P < 0.01; ***P < 0.001.

To determine whether the phenomenon of fibrosis-memory exists in mice with renal fibrosis, we assessed the impact of a first profibrotic renal injury on development of fibrosis during a second profibrotic injury of the contralateral kidney. We performed RUUO of the right kidney from day 0 to 6 (first injury), followed by an UUO of the left kidney from day 17 to 22 (second injury) (Supplemental Figure 1A; supplemental material available online with this article; https://doi.org/10.1172/JCI192095DS1, RUUO+UUO model). As control, the first injury on the right kidney was omitted or sham surgery was performed. Nonobstructed naive kidneys served as negative controls and showed no signs of fibrosis. Analysis of the left UUO kidney on day 22 revealed that a preceding fibrotic injury of the right kidney aggravated subsequent development of fibrosis in the left kidney (fibrosis-memory). Deposition of collagen-1 was increased by about 65%, and overall fibrosis quantified by Masson’s trichrome staining was increased by about 53%. There was also a significant increase in col1a1 mRNA expression (Figure 1A).

We then investigated whether the profibrotic priming persists for prolonged periods and compared 2 groups with different time intervals between RUUO of the right kidney and UUO of the left kidney. RUUO was performed from day 0 to 6 and UUO either from day 11 to 16 or from day 37 to 42. In control mice, no first injury was inflicted. Both groups with a preceding fibrosis on the right kidney developed significantly more fibrosis on the left kidney. There was no significant difference between the shorter and longer time interval (Figure 1, B and C), indicating that fibrosis-memory persists for several weeks.

We also quantified immune cells infiltrating the left UUO kidney by flow cytometry and found significantly more CD4^+^ T cells, CD8^+^ T cells, and monocytes in the UUO kidneys of mice with a preceding renal fibrosis (fibrosis-memory) (Figure 1D). UUO kidneys of mice with fibrosis-memory not only contained two times more infiltrating CD4^+^ T cells, but also a much higher percentage and absolute number of CD62L^–^CD69^+^ tissue-resident memory CD4^+^ T cells (CD4^+^ TRM cells) (Figure 1E and Supplemental Figure 2, A and B). Expression of CD103, CD279, and CD44 showed no or only small differences in UUO kidneys of mice with or without a preceding fibrotic event (Supplemental Figure 2C). No differences were detectable for basophils, eosinophils, and mast cells (Supplemental Figure 2D). UUO kidneys and spleens of mice with fibrosis-memory did not contain increased numbers of FoxP3^+^ regulatory T cells (Supplemental Figure 2E), and there was only a minimal expression of the exhaustion markers TIM-3 and LAG-3 on renal and splenic CD4^+^ T cells (Supplemental Figure 2F). The percentage of CD4^+^ TRM cells was also higher in the spleens of mice with fibrosis-memory (Figure 1E), while there was no increase in CD4^+^ T cells or CD4^+^ TRM cells in the livers or lungs and no development of fibrosis in liver, lung, or skin (Supplemental Figure 2, G and H). In summary, immunophenotyping (gating in Supplemental Figure 3) showed that there is more leukocyte infiltration during the second insult, suggesting that immune cells could be responsible for the enhanced fibrosis during the second insult.

To get more insight how a preceding RUUO may potentially influence the contralateral kidney before ureteral ligation of the contralateral kidney, we performed only a RUUO from day 0 to 6 and analyzed the contralateral kidneys on day 16. In comparison to kidneys from completely naive mice, we did not detect fibrosis or inflammation in the contralateral kidneys (Supplemental Figure 4, A and B). In a previous paper, we also studied how a UUO of one kidney influences the renal function (glomerular filtration rate [GFR]) of the contralateral kidney. Due to hyperfiltration, the GFR of the contralateral kidney transiently increased on day 1 after UUO (from 604 μL/min to 887 μL/min) but declined almost to basal levels afterward (706 μL/min on day 8; 633 μL/min on day 23) (12). Thus, at the time point when UUO of the left kidney was performed in the RUUO+UUO model, the left kidney was largely comparable to naive kidneys.

We also analyzed how fibrosis and inflammation in the RUUO kidney decline following reversal of the RUUO on day 6 (Supplemental Figure 4, A and B). As expected, there was a pronounced inflammation and fibrosis in the RUUO kidney, with infiltrating CD4^+^ T cells, neutrophils, and monocytes on day 6. On day 16, fibrosis was reduced by about 50%, while the leukocyte infiltrate in the RUUO kidney almost completely disappeared and was comparable to that of naive mice. In the spleens of mice with RUUO, we found increased numbers of neutrophils and monocytes on day 6, which fully normalized until day 16. In summary, the inflammatory response in the spleen and RUUO kidney had almost completely subsided at the time just prior to induction of the second renal injury.

### CD4^+^ T cells contribute to fibrosis-memory.

**Figure 2:**
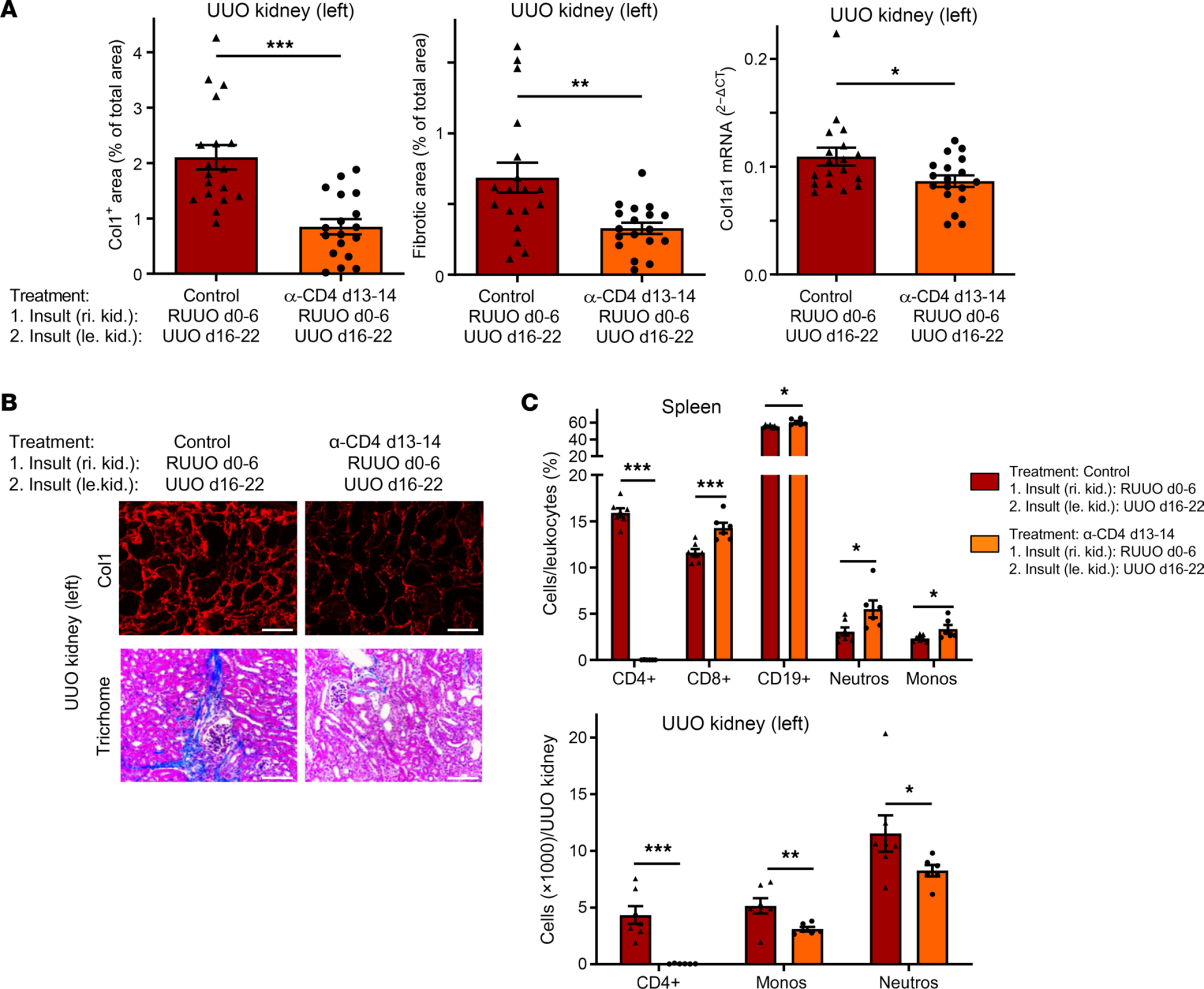
Reduced fibrosis-memory in the absence of CD4^+^ T cells. Mice underwent RUUO of the right kidney from day 0 to 6 (first insult), followed by UUO of the left kidney from day 16 to 22 (second insult). On days 13 and 14, mice were injected with a depleting CD4 antibody (α-CD4) or an isotype control antibody (control). (**A**) Quantification of collagen-1 (Col1), overall fibrosis (fibrotic area), and col1a1 mRNA expression in the left UUO kidney on day 22. Four pooled experiments, with *n* = 18/group. Controls are identical to those in Figure 6A. (**B**) Representative images of collagen-1 immunofluorescence and Trichrome staining. (**C**) Quantification of CD4^+^ T cells, CD8^+^ T cells, B cells (CD19^+^), monocytes (Monos), and neutrophils (Neutros) in the spleen and left UUO kidneys by flow cytometry (single experiment with *n* = 6–7/group). Scale bars: 100 μm. Data are represented as mean ± SEM. Unpaired 2-sided *t* test. *P < 0.05; **P < 0.01; ***P < 0.001.

To analyze whether fibrosis-memory is mediated by CD4^+^ T cells, we performed RUUO of the right kidney from day 0 to 6, depleted CD4^+^ T cells on day 13 and 14 with an anti-CD4 antibody, and quantified development of fibrosis during a subsequent UUO of the left kidney from day 16 to 22. As control, an isotype control antibody was injected, resulting in no depletion of CD4^+^ T cells. Without depletion of CD4^+^ T cells, a strong fibrosis developed in the left kidney, as seen before. In contrast, depletion of CD4^+^ T cells markedly reduced development of fibrosis in the left kidney by 60% for collagen-1, by 46% for overall fibrosis, and by 20% for collagen-1 mRNA expression (Figure 2, A and B). To verify that depletion of CD4^+^ T cells was effective and persisted until day 22, we quantified leukocyte subpopulations in the spleen and left UUO kidney on day 22 (Figure 2C). Almost no CD4^+^ T cells were detectable in the spleens and UUO kidneys of anti-CD4 treated mice on day 22. Depletion of CD4^+^ T cells also reduced the infiltration of monocytes and neutrophils in the UUO kidney on day 22.

These data suggest that increased fibrosis and inflammation in a fibrosis-memory recall situation involves CD4^+^ T cells.

### Adoptive transfer of CD4^+^ T cells from fibrotic donor mice increases fibrosis in the recipients.

**Figure 3:**
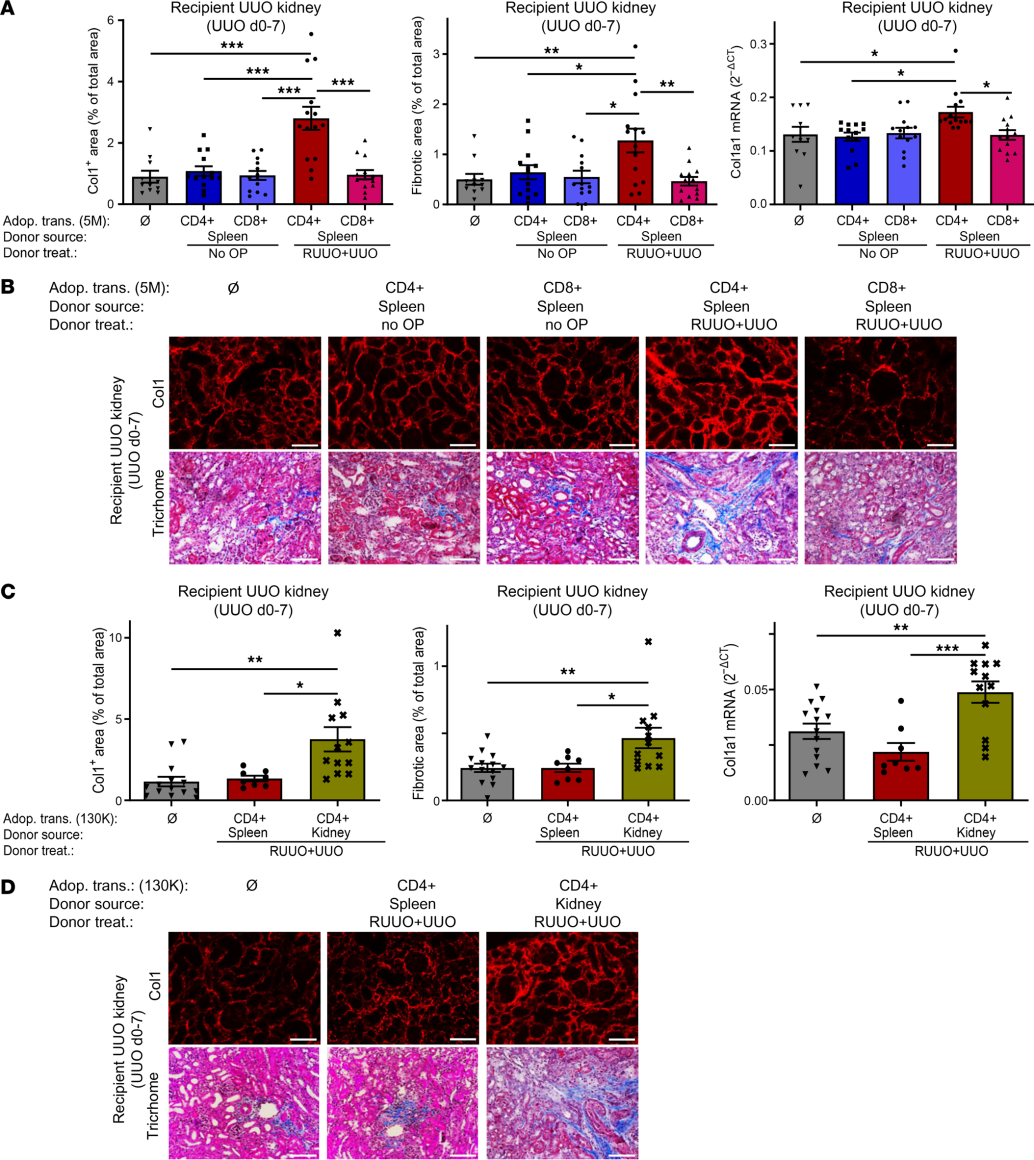
Fibrosis-memory can be adoptively transferred by splenic and renal CD4^+^ T cells. (**A** and **B**) CD4^+^ and CD8^+^ T cells were purified on day 0 from the spleens of naive donor mice (no OP) or donor mice after RUUO of the right kidney from day –18 to –12 and UUO of the same kidney from day –7 to 0 (RUUO+UUO). Where indicated, RPMI-1640 medium (Ø) or 5 × 10^6^ CD4^+^ T cells or CD8^+^ T cells were adoptively transferred into recipients on day 0 and UUO was performed on all recipients on day 0. (**A**) Quantification of collagen-1 (Col1), overall fibrosis (fibrotic area), and col1a1 mRNA expression in the recipient’s UUO kidneys on day 7. Two pooled experiments with *n* = 11, no cell transfer; 13, CD4^+^ T cells from naïve mice; 13, CD8^+^ T cells from naïve mice; 14, CD4^+^ T cells from mice after RUUO+UUO; and 13, CD8^+^ T cells from mice after RUUO+UUO. (**B**) Representative images of collagen-1 immunofluorescence and Trichrome staining. (**C** and **D**) CD4^+^ T cells were purified on day 0 from the spleens or fibrotic kidneys of donor mice, with renal fibrosis induced as described above (RUUO+UUO). Where indicated, medium or 130,000 CD4^+^ T cells were adoptively transferred into recipients on day 0 and UUO was performed on all recipients on day 0. (**C**) Analysis of the recipient’s UUO kidneys on day 7. Two pooled experiments with *n* = 14, no cell transfer; 8, CD4^+^ T cells from the spleen; and 12, CD4^+^ T cells from the UUO kidney. (**D**) Representative images of collagen-1 immunofluorescence and Trichrome staining. Scale bars: 100 μm. Data are represented as mean ± SEM. One-way ANOVA with multiple comparisons (**A** and **C**). *P < 0.05; **P < 0.01; ***P < 0.001.

To further show that CD4^+^ T cells are essential for the fibrosis-memory effect, we adoptively transferred T cells from fibrotic donor mice into naive recipients and performed UUO in the recipients (schematic overview in Supplemental Figure 1B). Two fibrotic injuries (RUUO+UUO) were inflicted on the right kidney of the donors (RUUO from day –18 to –12 and UUO from day –7 to 0) to induce a strong fibrosis-memory. CD4^+^ and CD8^+^ T cells were purified from the spleens of the fibrotic donors and adoptively transferred into naive recipients (5 × 10^6^ cells / recipient) immediately after the UUO operation of the recipients. Fibrosis was quantified 7 days later. In the control group, splenic CD4^+^ and CD8^+^ T cells of naive nonfibrotic donors were adoptively transferred or no adoptive transfer of cells was performed. Our data show that adoptive transfer of CD4^+^ T cells from the fibrotic, but not from the naive, donors markedly aggravated development of fibrosis in the UUO kidneys of the recipients (Figure 3A). CD8^+^ T cells were unable to transfer fibrosis-memory. Fibrosis was quantified as before by collagen-1 immunofluorescence, trichrome staining, and col1a1 mRNA. Representative images are shown in Figure 3B.

Next, we addressed the question whether renal CD4^+^ T cells from fibrotic donors are more profibrotic than splenic CD4^+^ T cells. Fibrosis was induced in the donor mice, as described above. As we could only obtain low numbers of CD4^+^ T cells from fibrotic kidneys, we adoptively transferred 130,000 cells/recipient. In the control group, no cells were transferred. 130,000 CD4^+^ T cells from fibrotic kidneys markedly enhanced fibrosis in the recipients, while the identical number of splenic CD4^+^ T cells from the same fibrotic donors had no effect (Figure 3, C and D).

### Fibrosis-memory leads to a strong clonal expansion of CD4^+^ T cells in the kidney and expands IL-3–producing memory T cells in the spleen and kidney.

**Figure 4:**
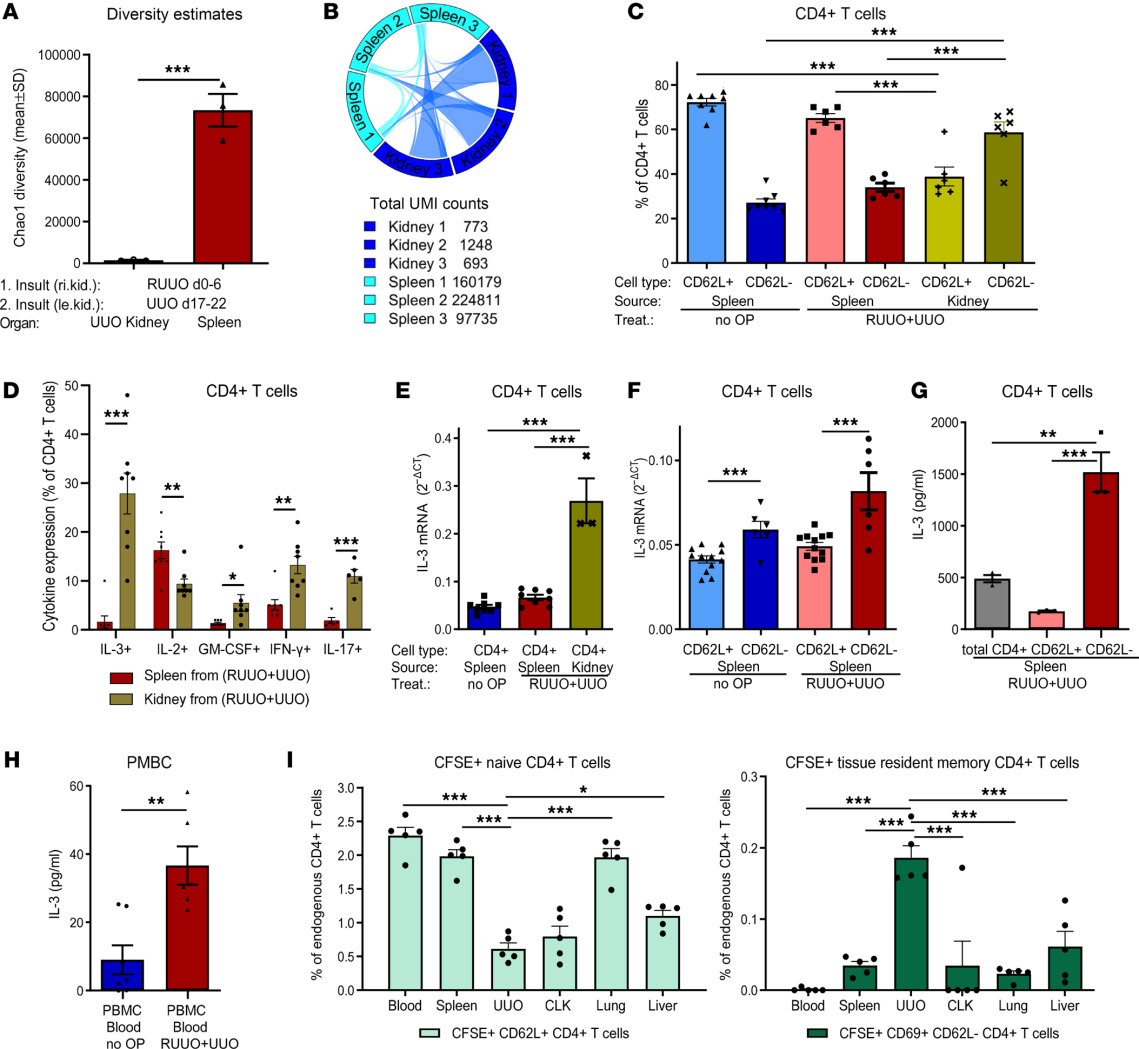
Fibrosis-memory leads to clonal expansion of CD4^+^ T cells in the kidney and increased IL-3 production. (**A** and **B**) RUUO of the right kidney (day 0–6), followed by UUO of the left kidney (day 17–22). (**A**) Clonal diversity of UUO kidney and splenic CD4^+^ T cells on day 22. (**B**) TCR repertoire overlaps between UUO kidney and splenic CD4^+^ T cells. UMI counts represent individual TCR-β mRNAs. (**C**–**I**) RUUO of the right kidney (day 0–6) and UUO of the same kidney (day 11–18) (RUUO+UUO). Analysis on day 18. Naive mice (no OP) served as controls. (**C**) CD62L^+^ and CD62L^–^ CD4^+^ T cells in the spleens of naive mice (*n* = 8), and in the spleens and fibrotic kidneys of RUUO+UUO mice (*n* = 6). (**D**) CD4^+^ T cells expressing IL-3, IL-2, GM-CSF, IFN-γ (*n* = 8 each), and IL-17 (*n* = 5) in the spleens and fibrotic kidneys of RUUO+UUO mice. (**E**) IL-3 mRNA expression of CD4^+^ T cells from the spleens of naive and RUUO+UUO mice (*n* = 8) and the fibrotic kidney of RUUO+UUO mice (*n* = 3). (**F**) IL-3 mRNA expression of CD62L^+^CD4^+^ T cells (*n* = 12) and CD62L^–^CD4^+^ T cells (*n* = 6) from the spleens of naive and RUUO+UUO mice. (**G**) IL-3 in the supernatant of anti-CD3–activated CD4^+^ T cells, CD62L^+^CD4^+^, and CD62L^–^CD4^+^ T cells from the spleens of RUUO+UUO mice (*n* = 3 per group). (**H**) IL-3 in the supernatant of anti-CD3–activated PBMCs from the blood of RUUO+UUO mice (*n* = 6) or naive mice (*n* = 7). (**I**) CFSE-labeled CD4^+^ T cells from the spleens of RUUO+UUO mice were injected into recipients 3 days after UUO operation (5 × 10^6^ cells/mouse, *n* = 5). Two days later, CFSE^+^ T cells were quantified in relation to endogenous CFSE^–^ CD4^+^ T cells in various organs. Data are represented as mean ± SEM. Unpaired 2-sided *t* test (**A**, **D**, **F**, and **H**) and 1-way ANOVA with multiple comparisons (**C**, **E**, **G**, and **I**). *P < 0.05; **P < 0.01; ***P < 0.001.

Having shown that CD4^+^ T cells mediate fibrosis-memory, we were interested in if there is a clonal expansion of CD4^+^ T cells in the UUO kidneys of mice with fibrosis-memory compared with the spleens of the same mice. Sequencing of the TCR-β chain showed that the clonal diversity of CD4^+^ T cells was much lower in the kidney than in the spleen (Figure 4A). In addition, TCR-β clonotypes of CD4^+^ T cells in the kidney of individual mice were to a large extent also present in the spleens of corresponding mice (Figure 4B). Overall, this indicates a strong oligoclonal expansion of CD4^+^ T cells in the fibrotic kidney, arguing for an autoreactivity of the infiltrating T cells against damaged/fibrotic renal tissue. To gain more insight into the phenotype of CD4^+^ T cells of fibrotic mice, we analyzed CD4^+^ T cells from the spleens and fibrotic kidneys of mice with RUUO+UUO, both performed on the right kidney. Naive mice without fibrosis served as controls. On day 18, naive and memory CD4^+^ T cells were identified by flow cytometry using CD62L as marker for naive (CD62L^+^) and memory (CD62L^–^) T cells (gating in Supplemental Figure 5A). In the fibrotic kidneys, about 60% of all CD4^+^ T cells had a memory phenotype, which was significantly higher than in the spleen, indicating that activated CD4^+^ T cells predominantly accumulate in the kidney (Figure 4C). Kidneys of naive mice contained very low numbers of T cells and were not included in this analysis.

In models of auto- and alloimmunity IL-3 was shown to play a major role for development of fibrosis (17–19). IL-3 is almost exclusively produced by T cells and thus, appeared as potential effector molecule to mediate T cell–dependent fibrosis-memory. We therefore analyzed the cytokine profile of CD4^+^ T cells in the spleens and UUO kidneys of mice with fibrosis-memory by intracellular cytokine staining (Figure 4D and Supplemental Figure 5, B and C). In the spleen, less than 2% of the CD4^+^ T cells expressed IL-3, while in the UUO kidney almost 30 % of the T cells expressed IL-3. There was also some enrichment of IFN-**γ–**, GM-CSF–, and IL-17–expressing CD4^+^ T cells in the UUO kidney, while IL-2–expressing T cells were less frequent in the kidney. We also analyzed which other cytokines were coexpressed by IL-3^+^CD4^+^ T cells. In the spleen, about 50% of the IL-3^+^ T cells coexpressed IL-2; however, in the kidney this coexpression was only 13%. IFN-**γ** and GM-CSF are only coexpressed by a minority of IL-3^+^ CD4^+^ T cells in both organs. Thus, IL-3^+^CD4^+^ T cells strongly accumulate in the kidneys of mice with fibrosis-memory. We also measured IL-3 mRNA expression in renal and splenic CD4^+^ T cells from fibrotic mice (RUUO+UUO). Consistent with intracellular cytokine staining, IL-3 mRNA expression was strongly upregulated in CD4^+^ T cells from fibrotic kidneys (Figure 4E). To quantify IL-3 expression in naive and memory CD4^+^ T cells, we purified these cells from the spleens of fibrotic (RUUO+UUO) or naive mice, induced cytokine expression with PMA/ionomycin, and quantified IL-3 mRNA expression by RT-PCR. We could not use flow cytometry because PMA/ionomycin interfered with detection of CD62L and other memory markers. Memory CD4^+^ T cells expressed higher IL-3-mRNA levels than naive CD4^+^ T cells, and IL-3 mRNA levels were further upregulated in memory CD4^+^ T cells of fibrotic mice (Figure 4F). Also on the protein level, CD62L^–^ memory CD4^+^ T cells from the spleens of RUUO+UUO mice secreted much more IL-3 than CD62L^+^ naive CD4^+^ T cells or total CD4^+^ T cells upon activation with anti-CD3 (Figure 4G). Increased IL-3 expression was also detectable in activated peripheral blood T cells of RUUO+UUO mice compared with naive mice (Figure 4H).

To investigate whether there is a preferential migration of certain CD4^+^ T cell subsets into the UUO kidney and to better understand the in vivo distribution of CD4^+^ T cells after adoptive transfer into mice with UUO, we adoptively transferred CD4^+^ splenic T cells labeled with carboxyfluorescein succinimidyl ester (CFSE) from fibrotic donor mice with RUUO+UUO into recipients with UUO. We have shown that adoptive transfer of such cells aggravated renal fibrosis in recipients with UUO (see Figure 3). CFSE-labeled cells were intravenously injected into recipients 3 days after UUO operation. Two days after injection, CFSE-labeled cells were quantified in the UUO kidney, the nonoperated contralateral kidney, blood, spleen, liver, and lung (Figure 4I). As the absolute numbers of CD4^+^ T cells are very different in the various organs, CFSE^+^ T cells were correlated to the endogenous (CFSE^–^) T cells present in each organ. CD4^+^ TRM T cells preferentially migrated into the UUO kidney and much less to other sites. In contrast, CFSE^+^ naive CD4^+^ T cells were preferentially found in the blood, spleen and lung.

### Adoptive transfer of memory CD4^+^ T cells from fibrotic donor mice increases fibrosis in recipients.

**Figure 5:**
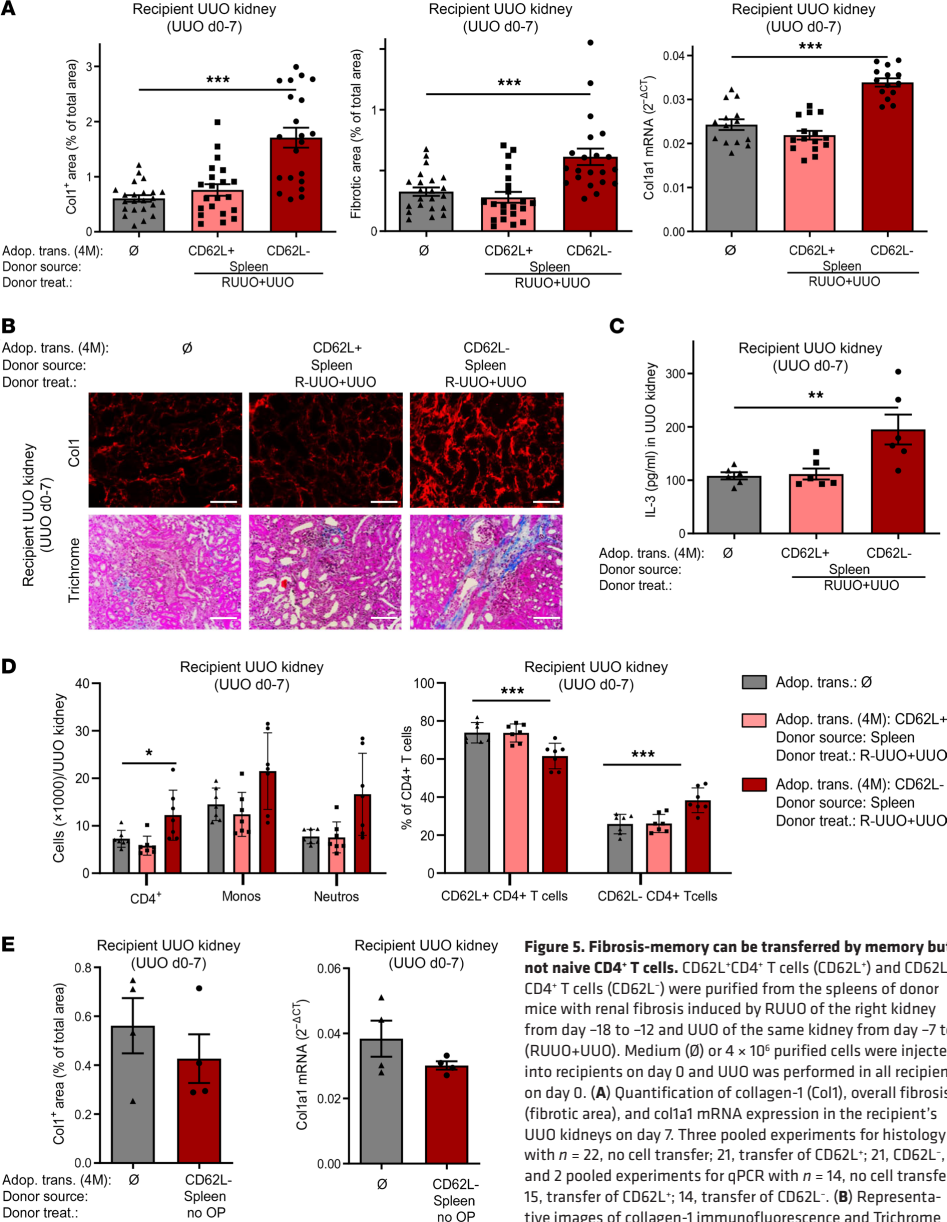
Fibrosis-memory can be transferred by memory but not naive CD4^+^ T cells. CD62L^+^CD4^+^ T cells (CD62L^+^) and CD62L^–^CD4^+^ T cells (CD62L^–^) were purified from the spleens of donor mice with renal fibrosis induced by RUUO of the right kidney from day –18 to –12 and UUO of the same kidney from day –7 to 0 (RUUO+UUO). Medium (Ø) or 4 × 10^6^ purified cells were injected into recipients on day 0 and UUO was performed in all recipients on day 0. (**A**) Quantification of collagen-1 (Col1), overall fibrosis (fibrotic area), and col1a1 mRNA expression in the recipient’s UUO kidneys on day 7. Three pooled experiments for histology with *n* = 22, no cell transfer; 21, transfer of CD62L^+^; 21, CD62L^–^, and 2 pooled experiments for qPCR with *n* = 14, no cell transfer; 15, transfer of CD62L^+^; 14, transfer of CD62L^–^. (**B**) Representative images of collagen-1 immunofluorescence and Trichrome staining. (**C**) Quantification of IL-3 in the supernatant of shredded recipient’s UUO kidneys (*n* = 6/group). (**D**) CD4^+^ T cells, monocytes (Monos), and neutrophils (Neutros) as well as percentage of CD62L^+^ and CD62L^–^CD4^+^ T cells in the recipient’s UUO kidneys on day 7 (*n* = 7/group). Scale bars: 100 μm. Data are represented as mean ± SEM. One-way ANOVA with multiple comparisons. *P < 0.05; **P < 0.01; ***P < 0.001.

To show that memory but not naive CD4^+^ T cells mediate fibrosis-memory, we purified CD62L^+^ or CD62L^–^ CD4^+^ T cells from the spleens of fibrotic donor mice (RUUO+UUO) (Supplemental Figure 5D), adoptively transferred the cells into naive recipients, and performed UUO in the recipients immediately before the T cell transfer. In the control group, no cells were transferred. Quantification of fibrosis in the recipient’s UUO kidneys revealed that the adoptive transfer of memory CD4^+^ T cells but not naive CD4^+^ T cells from fibrotic donor mice significantly enhanced fibrosis as shown by collagen-1 deposition, overall fibrosis, and col1a1 mRNA expression (Figure 5, A and B). Recipient UUO kidneys were shredded, and IL-3 was quantified in the cell supernatant by ELISA. IL-3 protein levels were significantly increased after adoptive transfer of memory but not naive CD4^+^ T cells from fibrotic donors (Figure 5C). The adoptive transfer of memory CD4^+^ T cells from fibrotic donors also increased the leukocyte infiltration in the recipient’s UUO kidney with more CD4^+^ T cells, a higher percentage of memory CD4^+^ T cells, more neutrophils, and, nonsignificantly, more monocytes (Figure 5D), similar to what we have observed previously in the RUUO+UUO model (Figure 1D). To find out if fibrosis-memory is a shared characteristic of memory CD4^+^ T cells in general or only of memory CD4^+^ T cells from fibrotic donors, we isolated CD62L^–^CD4^+^ memory T cells from the spleens of naive donor mice without fibrosis, adoptively transferred 4 × 10^6^ of these cells into naive recipients, and performed UUO in the recipients immediately before the T cell transfer (Figure 5E). In the control group, we used a group without T cell transfer. No enhancement of fibrosis was seen in the recipients of memory CD4^+^ T cells from donors without fibrosis, indicating that fibrosis-memory only develops in memory CD4^+^ T cells under fibrotic conditions.

### Enhancement of fibrosis in fibrosis-memory situations depends on IL-3.

In order to study the contribution of IL-3 for aggravation of fibrosis in fibrosis-memory situations we blocked IL-3 with an antibody and used IL-3–deficient mice.

**Figure 6:**
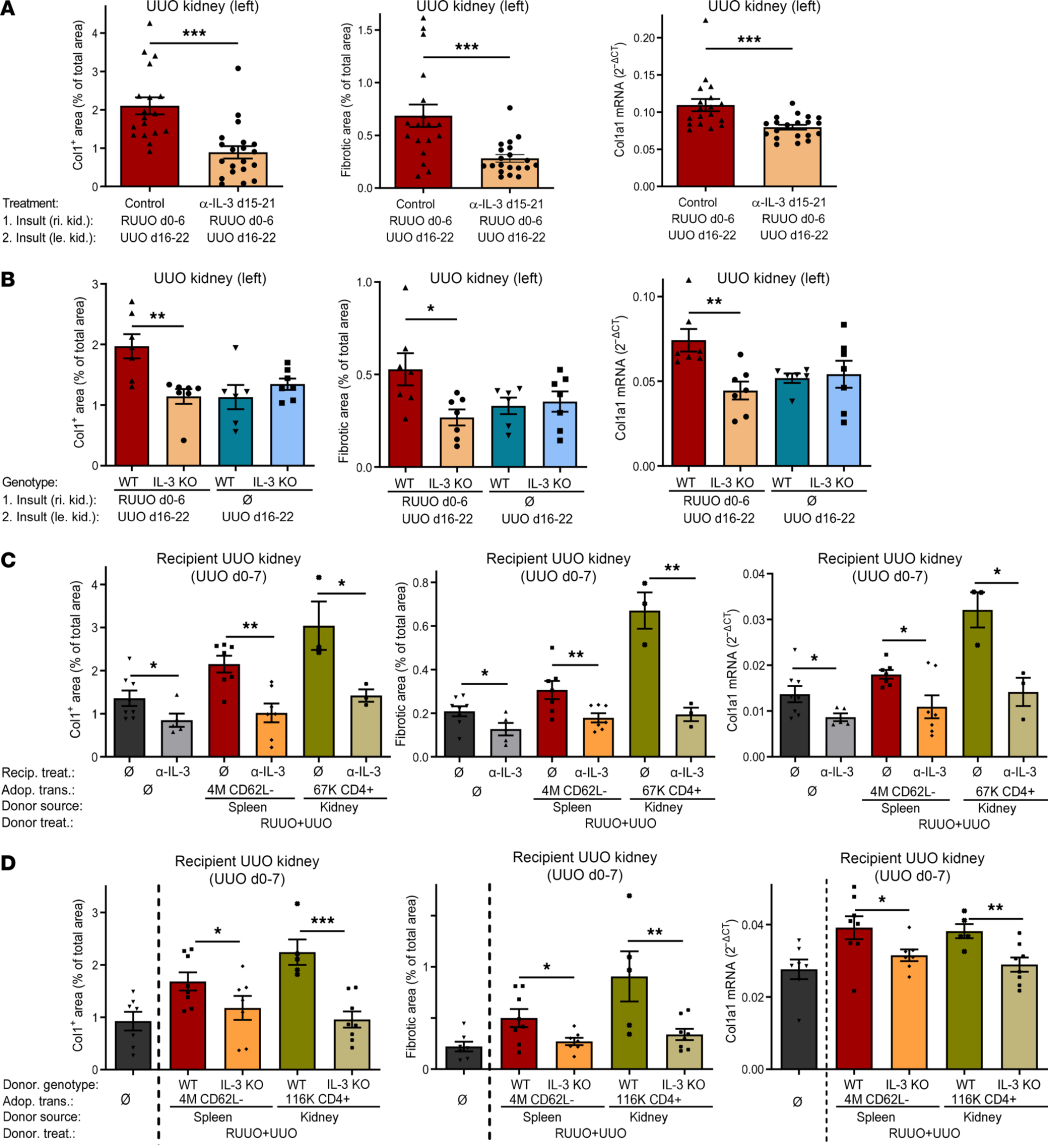
Retrieval and adoptive transfer of fibrosis-memory is dependent on T cell–derived IL-3. (**A**) RUUO of the right kidney (day 0–6, first insult) followed by UUO of the left kidney (day 16–22, second insult). Treatment with anti–IL-3 (*n* = 20) or isotype control antibody (*n* = 18; 4 pooled experiments, same controls as in Figure 2A) from day 15–21. Analysis of the left UUO kidneys on day 22. (**B**) WT or IL-3–KO mice underwent RUUO of the right kidney (day 0–6, first insult) or remained naive (Ø). All mice then underwent UUO of the left kidney (day 16–22). Analysis of the left UUO kidneys on day 22. One experiment with *n* = 7, WT RUUO+UUO; 7, IL-3-KO RUUO+UUO; 6, WT UUO; and 7, IL-3-KO UUO. (**C**) Splenic CD62L^–^CD4^+^ or renal CD4^+^ T cells were purified from the spleens or fibrotic kidneys of RUUO+UUO donor mice (RUUO from day –18 to –12 and UUO of the same kidney from day –7 to 0). Medium, 4 × 10^6^ splenic CD62L^–^CD4^+^ or 67,000 renal CD4^+^ T cells were injected into recipients on day 0. UUO in all recipients on day 0. Treatment with anti–IL-3 or an isotype control antibody (Ø) from day 0–6. Analysis of the recipient’s UUO kidneys on day 7. Single experiment with *n* = 8, no cell transfer; 5, no cell transfer and anti–IL-3; 7, transfer of CD62L^-^ from spleen; 7, transfer of CD62L^-^ from spleen and anti–IL-3; 3, transfer of CD4^+^ from UUO kidney; and 3, transfer of CD4^+^ from UUO kidney and anti–IL-3. (**D**) Splenic CD62L^–^CD4^+^ or renal CD4^+^ T cells were purified from the spleens or fibrotic kidneys of WT or IL-3–deficient (IL-3 KO) RUUO+UUO mice, as in **C**. Medium (Ø), 4 × 10^6^ splenic CD62L^–^CD4^+^ or 116,000 renal CD4^+^ T cells were injected on day 0. UUO in all recipients on day 0. Analysis of the recipient’s UUO kidneys on day 7. Single experiment with *n* = 7, no cell transfer; 8, CD62L^+^ from spleen of WT; 7, CD62L^+^ from spleen of IL-3 KO; 5, CD62L^+^ from UUO-kidney of WT; and 8, CD62L^+^ from UUO-kidney of IL-3 KO. Data are represented as mean ± SEM. Unpaired 2-sided *t* test. *P < 0.05; **P < 0.01; ***P < 0.001.

In the RUUO+UUO model, RUUO was performed on the right kidney from day 0 to 6 followed by UUO of the left kidney from day 16 to 22. A blocking IL-3 antibody or an isotype control antibody was injected daily from day 15 to 21. Analysis of the left UUO kidney on day 22 revealed that blockade of IL-3 during the second injury (recall period) markedly reduced development of fibrosis, as shown by collagen-1 deposition, overall fibrosis, and col1a1 mRNA expression (Figure 6A). In contrast, blockade of IFN-**γ** or GM-CSF during the second injury did not reduce development of fibrosis (Supplemental Figure 6A).

We also performed the same experiment with IL-3–deficient mice (Figure 6B). RUUO was performed on the right kidney of WT or IL-3–deficient mice from day 0 to 6 (first injury), followed by an UUO of the left kidney from day 16 to 22 (second injury). In one group of mice, the first injury was omitted and only the UUO of the left kidney was performed. Quantification of fibrosis in the left UUO kidney of WT and IL-3–KO mice revealed that development of fibrosis during the second insult was aggravated only in WT mice but not in IL-3–KO mice. Furthermore, deficiency of IL-3 reduced development of fibrosis only in a memory situation with a preceding fibrotic event, but not during the first insult, consisting of a 6-day UUO without a preceding fibrotic event.

### Adoptive transfer of fibrosis-memory is dependent on IL-3–producing CD4^+^ T cells.

To further show the contribution of IL-3 for aggravation of fibrosis in a fibrosis-memory situation, we studied the role of IL-3 in the adoptive transfer model (Figure 6C). CD4^+^ T cells were isolated from the spleens or fibrotic kidneys of mice with RUUO+UUO. 4 × 10^6^ splenic memory CD4^+^ T cells or 67,000 renal CD4^+^ T cells were adoptively transferred to naive recipients, and UUO was performed in the recipients immediately before adoptive cell transfer on day 0. In one group, no adoptive cell transfer was performed. In the recipients, IL-3 was blocked with a monoclonal antibody injected daily from day 0 to 6, and recipient UUO kidneys were analyzed on day 7. In the absence of anti–IL-3 adoptive transfer of splenic memory CD4^+^ T cells and renal CD4^+^ T cells markedly enhanced development of fibrosis in the recipients. In contrast, blockade of IL-3 completely abrogated the enhancement of fibrosis in the recipients (Figure 6C). In mice without adoptive transfer of T cells blockade of IL-3 had smaller antifibrotic effects. Consistent results were obtained with three different methods to quantify fibrosis.

To demonstrate that IL-3 derived from CD4^+^ T cells is essential for aggravation of fibrosis in a fibrosis-memory situation, we used WT and IL-3–deficient mice as donors of CD4^+^ T cells (Figure 6D). In the donors, fibrosis was induced with RUUO and UUO (RUUO+UUO) as described above. Adoptive transfer of splenic memory CD4^+^ T cells and renal CD4^+^ T cells from WT donors markedly aggravated development of fibrosis in the recipient UUO kidneys. Mice without transfer of T cells served as controls. In contrast, adoptive transfer of IL-3–deficient splenic or renal CD4^+^ T cells from fibrotic donors did not aggravate development of fibrosis in the recipient’s UUO kidneys (Figure 6D).

In summary, these data show that IL-3 released from memory CD4^+^ T cells is an essential factor for aggravation of fibrosis in a fibrosis-memory situation.

### Macrophages but not basophils or monocytes are potential profibrotic mediators of IL-3.

A recent publication showed that basophils contribute to fibrosis in the UUO model (20). As basophils strongly react to IL-3, they could be a potential link between IL-3 and fibrosis in the RUUO+UUO fibrosis-memory model. RUUO was performed from day 0 to 6 followed by UUO from day 16 to 22. Basophils were or were not depleted with the antibody MAR-1 from day 12 to 22. Despite complete depletion of basophils from the spleen and UUO kidney, there was no effect of basophil depletion on development of fibrosis in the UUO kidney on day 22 (Supplemental Figure 6, B and C). In addition, monocytes have been associated with development of fibrosis (21–25). Thus, we depleted CCR2^+^ monocytes with the well-established antibody MC-21 from day 16 to 22. Complete depletion of monocytes from the peripheral blood and the UUO kidney had no effect on development of fibrosis in the UUO kidney on day 22 (Supplemental Figure 7), consistent with the observation that Ly6C^+^ monocytes did not express IL-3R in the UUO kidney (Supplemental Figure 8A). Apart from infiltrating classical monocytes, the kidney also contains various subsets of interstitial macrophages. We thus performed flow cytometry on single-cell suspensions of UUO kidneys to study the expression of the IL-3 receptor (IL-3R) CD123. We found a high expression of CD123 on CD206^+^ F4/80-high macrophages, which are strongly associated with development of fibrosis in the UUO model (26, 27) (Supplemental Figure 8, A and B). Thus, IL-3R–expressing CD206^+^ macrophages could be potential downstream mediators of IL-3–dependent fibrosis.

### T cell–dependent expression of IL-3 is increased in patients with SSc.

**Figure 7:**
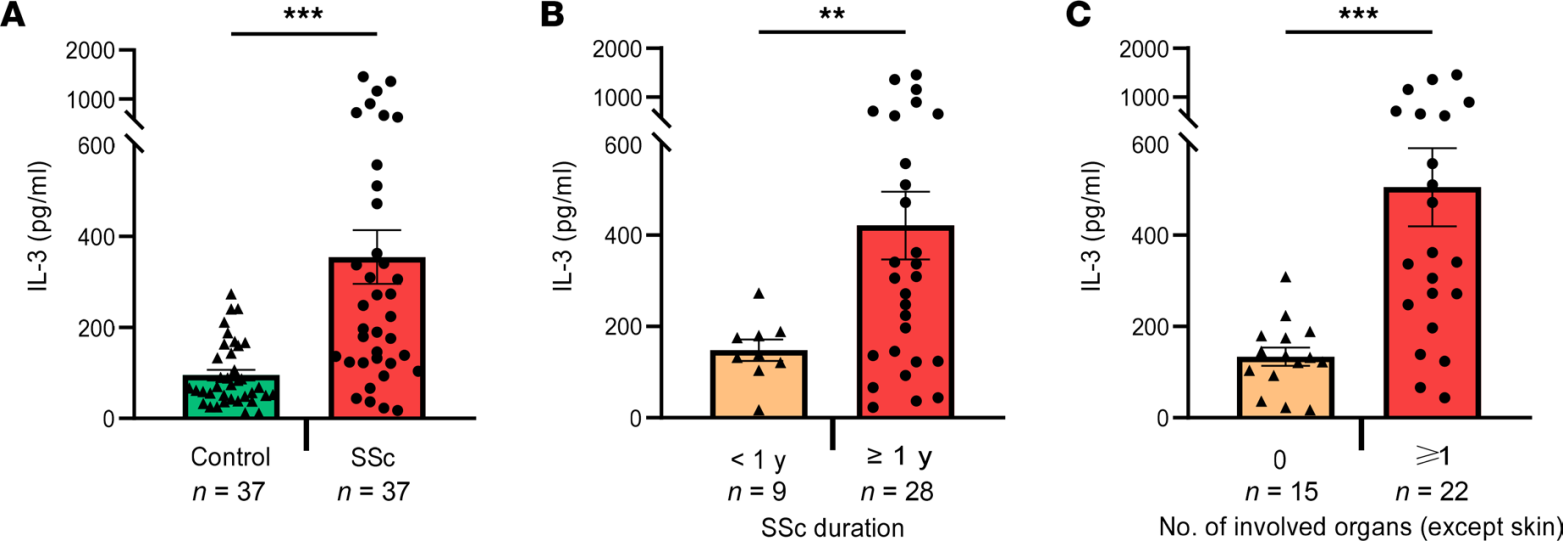
Increased T cell–dependent expression of IL-3 in patients with systemic sclerosis. PBMCs from patients with systemic sclerosis (SSc) (*n* = 37) and individuals acting as healthy controls (*n* = 37) were cultured with anti-CD3 for 3 days. IL-3 expression was measured in the cell culture supernatant by ELISA. (**A**) IL-3 expression in individuals acting as healthy controls and patients with SSc. (**B**) IL-3 expression in patients with SSc stratified according to disease duration. (**C**) IL-3 expression in patients with SSc stratified according to organ involvement (skin was not counted as an organ). Data are represented as mean ± SEM. Welch’s 2-sided *t* test. **P < 0.01; ***P < 0.001.

In order to translate the data to humans, we analyzed IL-3 expression in patients with SSc, a fibrotic disease with limited therapeutic options. Patient characteristics are shown in Table 1. SSc always manifests at the skin but can also involve internal organs. IL-3 expression was quantified in the supernatant of PBMCs cultured with anti-CD3 for 3 days to induce T cell activation and to stimulate the release of IL-3. Patients with SSc (*n* = 37) showed a significantly higher IL-3 expression than individuals acting as healthy controls (*n* = 37) (Figure 7A). Patients with SSc were also stratified according to disease duration below or above 1 year and according to mere skin involvement or additional organ fibrosis. Remarkably, a much higher level of IL-3 expression was found in patients with disease duration above 1 year (Figure 7, B and C), suggesting that fibrosis-memory has built up after prolonged disease duration. Spreading of fibrosis to one or more internal organs was also associated with a higher IL-3 expression.

## Discussion

Our data provide evidence for the generation of fibrosis-memory in a model of renal fibrosis and show that fibrosis-memory is a relevant enhancer of fibrosis during a second renal injury. Fibrosis-memory was dependent on IL-3–producing memory CD4^+^ T cells. IL-3–expressing CD4^+^ T cells and tissue-resident memory T cells were strongly enriched in the fibrotic kidney after a second insult compared with the spleen along with a strong clonal expansion of CD4^+^ T cells in the fibrotic kidney. Expansion and activation of IL-3–expressing memory T cells in the kidney was most likely triggered by release of autoantigens and retroviral elements from damaged renal cells (28). Damage-associated autoantigens might be kidney-specific, resulting in a kidney-specific fibrosis-memory or damage-memory. Alternatively, these autoantigens might be more universal and could activate T cells also in other tissues upon cell damage. We have not tested whether renal fibrosis or adoptive transfer of IL-3–expressing memory T cells from fibrotic donors enhance development of fibrosis in other damaged organs like liver or lung. However, we found that renal fibrosis-memory did not spread to the undamaged liver or lung and did not induce CD4^+^ T cell migration to these organs, consistent with our observation that transferred CD4^+^ memory T cells from fibrotic donors preferentially migrated into the damaged UUO kidneys and much less to other sites without damage.

IL-3 has been recognized as a profibrotic molecule in models of chronic autoimmunity and allograft rejection, such as MLR-lpr lupus nephritis (19), autoimmune myocarditis (17), and chronic heart transplant rejection (18). In all of these rather chronic models, fibrosis was quantified only after several weeks, when fibrosis-memory had time to build up and contribute to fibrosis. Experiments with IL-3–KO mice showed that IL-3 has no role for development of fibrosis during the first profibrotic damage to the kidney (Figure 6B, blue bars), while a neutralizing anti–IL-3 antibody reduced development of fibrosis also during the first damage (Figure 6C, gray bars). Developmental effects and compensatory mechanisms in global IL-3 KO mice may explain this discrepancy. Adoptive transfer of CD4^+^ T cells together with blockade of IL-3 in the recipients (Figure 6C) as well as blockade of IL-3 during the second insult (Figure 6B) clearly showed that T cell–derived IL-3 is an essential effector molecule of fibrosis-memory.

Although IL-3 is mainly expressed by T cells (29), some IL-3 expression has also been found by B cells and basophils and locally in the brain by astrocytes and possibly neurons and microglia (30–34). Our data show that IL-3 derived from memory T cells largely explains the phenomenon of fibrosis-memory in the RUUO+UUO model.

Downstream effects of IL-3 are rather restricted, as the IL-3R CD123 is expressed only by small subsets of cells. Basophils strongly express the IL-3R (35) and have been linked to development of fibrosis in models of allograft fibrosis, renal fibrosis (UUO), and myocardial infarction (20, 36, 37). However, in our fibrosis-memory model, we found only very few basophils in the kidney and no role of basophils for development of fibrosis. Apart from basophils, human but not mouse pDCs strongly express IL-3R (38). Numerous publications have shown an important role of pDCs for development of fibrosis, considering CXCL4 as a main profibrotic mediator of pDCs (39–42). IL-3R is also expressed on nonclassical CD16^+^ human monocytes and can be upregulated on classical monocytes by various cytokines (43). Monocytes are well known to be involved in development of fibrosis and wound healing (21–25) and may also directly contribute to production of collagen-1 (12, 44–46). In addition, it was suggested that they are involved in IL-3–dependent myocardial fibrosis (17). However, depletion of CCR2^+^Ly6C^+^ classical monocytes did not reduce fibrosis in the RUUO+UUO model. In contrast, we found strong IL-3R expression on F4/80-high macrophages that are strongly associated with development of fibrosis in the UUO model (26, 27). Thus, IL-3R^+^ macrophages appear as possible downstream mediators of IL-3–dependent fibrosis.

We observed that fibrosis-memory established either by a preceding fibrotic event or by adoptive transfer of memory CD4^+^ T cells from fibrotic donors was associated with an enhanced leukocyte infiltration in the damaged kidney. It is known that human and murine endothelial cells express IL-3R and, upon exposure to IL-3, upregulate the adhesion molecules E- and P- selectin (CD62E and CD62P) (47–49). Transendothelial migration of leukocytes into inflamed CNS tissue was shown to be strongly dependent on IL-3 (50). Thus, IL-3 released by profibrotic memory T cells might also contribute to the enhanced inflammation observed during fibrosis-memory. Most likely, multiple different IL-3R^+^ cells contribute to IL-3–mediated enhanced fibrosis and inflammation, with none of them having a dominant, nonredundant role.

We found that renal CD4^+^ T cells are more effective in transferring fibrosis-memory than splenic T cells and that the percentage of memory CD4^+^ T cells and their expression of IL-3 were much higher in renal compared with splenic T cells. However, profibrotic memory T cells are also present in the spleen, as adoptive transfer of fibrosis-memory was possible with larger numbers splenic CD4^+^ T cells. Indeed, CD4^+^ T cells with a tissue-resident memory phenotype (CD62L^–^CD69^+^) were increased in the spleens of mice with fibrosis-memory, suggesting that there is some exchange of profibrotic memory T cells between kidney and spleen. It seems that renal CD4^+^ TRMs can migrate into the spleen after damage-induced activation in the UUO kidney (Figure 1E). Adoptive transfer experiments suggest that CD4^+^ TRMs reach the contralateral kidney, if damage is induced there (Figure 4I, right), although it cannot be fully excluded that the CD4^+^ T cells change their phenotype after adoptive transfer. The first fibrotic insult in one kidney does not increase the number of CD4^+^ T cells in the undamaged contralateral kidney compared with kidneys from completely naive mice (Supplemental Figure 4B, left), indicating that there is little migration of CD4^+^ T cells from a damaged kidney into the undamaged contralateral kidney. There was only some increase of monocytes in the undamaged contralateral kidney; however, depletion of monocytes did not interfere with aggravation of fibrosis during a second insult (Supplemental Figure 7), arguing against a role of monocytes in a fibrosis-memory situation.

Analysis of patients with SSc revealed a marked upregulation of IL-3 expression after prolonged disease duration and after spreading of fibrosis to several organs, suggesting that fibrosis-memory has built up over time and may contribute to the progression of fibrosis from a localized to a systemic stage. Our data show that CD4^+^ memory T cells enhance fibrosis in memory situations; however, inhibition of memory T cells by conventional immunosuppressive drugs is known to be difficult in clinical settings, as conventional drugs like calcineurin-inhibitors or costimulation blockers mainly act on naive and less on memory T cells. Blockade of IL-3 derived from memory T cells could be a specific and effective approach to interfere with fibrosis-memory and might overcome current limitations in inhibiting memory T cells. Overall, our functional studies in mice and translational findings in patients suggest that blockade of IL-3 might be a promising strategy to interfere with deleterious effects of fibrosis-memory and to treat chronic progressive fibrotic diseases.

## Methods

### Sex as a biological variable.

For the human study, both sexes were involved without selection. All animal experiments were performed with female mice at an age of 10–14 weeks to reduce heterogeneity within groups.

### Mice.

All mice were bred and housed under specific pathogen–free conditions in the animal facility of the University Hospital Regensburg. Food and water were provided ad libitum. WT C57BL/6 mice were purchased from Janvier Labs. IL-3–deficient mice (C.129S2(B6)-Il3<tm1Glli>/Rbrc) (51) on a C57BL/6 background were obtained as described previously (50).

### Murine model of renal fibrosis.

Mice were anesthetized by intraperitoneally injection of medetomidine (0.5 mg/kg), midazolam (5 mg/kg), and fentanyl (0.05 mg/kg). Anesthesia was reversed by s.c. administration of atipamezole (2.5 mg/kg), flumazenil (0.5 mg/kg), and naloxone (1.2 mg/kg). Tramadol (0.1 mg/mL) in drinking water was started immediately after surgery and continued for 2 days postoperatively. In addition, the animals were treated with 200 mg/kg paracetamol s.c. and 25 mg/kg tramadol s.c. immediately before surgery. No antibiotics were given to the mice.

To achieve UUO, the right ureter was ligated through a low midline abdominal incision on day 0. On day 7 after UUO, kidneys were harvested and analyzed. RUUO was performed as previously described (12). To ensure reversibility, a displaceable clip (Fine Science Tools) was used. This clip was placed on the right ureter on day 0, shifted in position on days 2 and 4 to avoid strictures, and removed on day 6. For controls, sham surgery was performed without placing a clip. Mice underwent RUUO of the right kidney from day 0 to 6, followed by UUO of the left kidney from day 16 to 22. Prior to UUO of the left kidney, specific immune cells or cytokines were depleted by intraperitoneal injections of antibodies. Equivalent amounts of isotype control antibodies were used as control. CD4^+^ T cells were depleted with 1 mg anti-CD4 antibody (clone GK1.5, BioXCell) on day 13 and 0.5 mg GK1.5 on day 14. Control mice received rat IgG2b (clone LTF-2, BioXCell). Basophils were depleted with 10 μg anti-FcεR1α antibody (clone MAR-1, eBiosciences) from day 12 to 14. Control mice received Syrian Hamster IgG (RRID AB_2337026, Jackson ImmunoResearch Europe). Monocytes were depleted with 75 μg anti-CCR2 antibody (clone MC-21) from day 16 to 21 (46). Control mice received rat IgG2b isotype control antibody (clone LTF-2, BioXCell). Complete depletion of the respective cell types was maintained until the end of the experiment on day 22. To block IL-3, mice received daily intraperitoneal injection of 75 μg anti–IL-3 (clone MP2-8F8, BioXCell) from day 15 to day 21. Control mice received rat IgG1 isotype antibody (clone HRPN, BioXCell). For GM-CSF blockade, mice received 200 μg anti-GM-CSF (clone MP1-22E9, BioLegend); for IFN-**γ** blockade, mice received 200 μg anti–IFN-**γ** (clone XMG1.2, BioLegend) on days 15, 17, 19 and 21. Control mice received rat IgG2a isotype control antibody (clone RTK2758, BioLegend).

### Isolation of T cells, adoptive cell transfer, and culture of T cells.

Donor mice were either naive or underwent RUUO of the right kidney from day –18 to –12, followed by UUO of the same kidney from day –7 to 0 (RUUO+UUO). T cells were isolated from the spleens of nonoperated donor mice and spleens or kidneys of operated donor mice on day 0. Splenic CD4^+^ or CD8^+^ T cells were isolated with CD4 or CD8 microbeads (Miltenyi). For isolation of splenic CD62L^+^CD4^+^ and CD62L^–^CD4^+^ T cells, the CD4^+^ T cells were first isolated with a CD4^+^ T cell negative isolation kit (Miltenyi) and subsequently separated with CD62L microbeads (Miltenyi). CD4^+^ T cells were isolated from the kidneys of RUUO+UUO donor mice in two steps: first with a CD4^+^ T cell negative isolation kit (Miltenyi) and then with CD4 (L3T4) microbeads (Miltenyi). Separation of CD62L^+^ or CD62L^–^CD4^+^ T cells from the kidney was not feasible due to the low yield of cells. All isolations were performed according to the manufacturer’s protocol (Miltenyi Biotech). The purity of all cell populations was between 93% and 99%, as verified by flow cytometry, and the viability of the transferred cells was above 90% by trypan blue staining. Cell numbers of adoptively transferred cells relate to the number of viable cells. Adoptive transfer of cells was performed in RPMI-1640 medium by intravenous administration into recipients on day 0, and UUO was performed on the right kidney immediately before adoptive cell transfer. Analysis of recipient UUO kidneys was performed on day 7. Total, CD62L^+^, or CD62L^–^ CD4^+^ T cells from RUUO+UUO mice were cultured for 2 days in 96 wells coated with anti-CD3 antibodies (100,000 cells/well in 200 μL RPMI-1640 with 10% FCS, 1% penicillin/streptomycin, 2 mM L-pyruvat, 50 μM b-mercaptoethanol and 20 ng/mL mouse IL-2, Peprotech). Plates were coated with 5 μg/mL anti-CD3 at 37°C for 3 hours (clone 145-2C11, Biolegend) and subsequently washed two times with PBS.

### Cell staining and flow cytometry.

To prepare single-cell suspensions of spleen and kidney tissue, mice were sacrificed with carbon dioxide and transcardially perfused with 20 mL NaCl 0.9%. Spleens and kidneys were cut into small pieces and pressed through a 70 μm mesh followed by a 40 μm cell strainer. For staining of macrophages, UUO kidneys were cut into small pieces and digested with 1 mg/mL collagenase (Sigma-Aldrich, C0130) in 5 mL HANKs buffer (Sigma-Aldrich, H8265) for 30 minutes and pressed through a 70 μm mesh followed by a 40 μm cell strainer. Cells were stained for 20 minutes with a combination of directly labeled antibodies: anti-CD45-V500 (clone 30-F11), anti-CD4-PE-Cy5 (clone RM4-5), anti-CD11b-FITC (clone M1/70) purchased from BD Biosciences; anti-CD4-APC (clone RM4-4), anti-CD4-PerCp (clone RM4-5), anti-CD8a-APC-Cy7 (clone 53-6.7), anti-Ly6G-Pacificblue (clone 1A8), anti-Ly6C-PE-Cy7 (clone HK1.4), anti-CD44-APC-Cy7 (clone IM7), anti-CD62L-APC (MEL-14), anti-CD69-Pacificblue (clone H1.2F3), anti-CD123-PE (clone 5B11), anti-CD206-PE (clone C068C2), F4/80-APC (clone BM8), anti-MHCII-APC-Cy7 (clone M5/114.15.2), anti-LAG3-PE-Cy7 (clone C9B7W), anti-TIM3-PE (clone RMT3-23) purchased from BioLegend; anti-FoxP3-PE (clone FJK-16s) purchased from Thermofisher/eBioscience. Red blood cells were lysed with FACS-lysing solution (BD Biosciences), and samples were acquired on a FACSCanto-II (BD Biosciences) and analyzed with FACSDiva software. Cell debris and dead cells were excluded by their light scatter properties, and leukocyte subsets were identified by light scatter properties and surface marker expression. The number of cells was quantified with counting beads (Invitrogen). For intracellular staining of IL-3, splenocytes and renal cells were activated with PMA (10 ng/mL), ionomycin (1 μg/mL), and brefeldin A (5 μg/mL) for 3 hours. After staining with anti-CD45-AmCyan (clone 30-F11) and anti-CD4-PerCp-Cy5.5 (clone RM4-5), cells were treated with Fix-Perm and Perm-Wash solutions (BD Biosciences) and stained intracellularly with anti-IFN-**γ**-FITC (clone XMG1.2), anti–IL-3-PE (clone MP2-8F8), anti–IL-2-PE-Cy7 (clone JES6-5h4), anti-GM-CSF-BV421 (clone MP1-22E9), and anti–IL-17 (clone 17B7).

### Quantification of cytokines and cell culture.

IL-3 was quantified in the supernatant of shredded renal tissue and in cell culture supernatant using an ELISA. To obtain the supernatant of shredded renal tissue, 1 kidney was cut into small pieces and pressed through a 70 μm mesh in a total volume of 500 μL PBS containing 1% BSA. The suspension was centrifuged at 10,621 × *g* for 10 minutes, and the supernatant was recovered for ELISA. For cell culture, splenic CD4^+^ T cells and CD62L^+^CD4^+^ and CD62L^–^CD4^+^ T cells were isolated as described above. PBMCs were prepared by Ficoll-Paque density gradient centrifugation from EDTA-anticoagulated fresh blood samples. Splenic cells were stimulated for 2 days, and PBMCs were stimulated for 3 days with anti-CD3 antibody (2 μg/mL, clone 145-2C11). The IL-3 concentration was measured in the supernatant by ELISA according to the manufacturer’s protocol (R&D Systems, DY403).

### RNA isolation and real-time PCR.

Total RNA was isolated from renal tissue with the NucleoSpin RNA Plus Kit (Macherey-Nagel). To obtain RNA from purified T cells, the cells were first activated with PMA (10 ng/mL), ionomycin (1 μg/mL), and brefeldin A (5 μg/mL) for 3 hours, and RNA was isolated with the NucleoSpin RNA Plus XS Kit (Macherey-Nagel). RNA was quantified by a NanoDrop spectrophotometer. Reverse transcription was performed using the Omniscript RT kit (Quiagen). Quantitative real-time PCR was performed with 2 ng reversely transcribed RNA using the QuantiTect SYBRGreen PCR Kit (Qiagen GmbH) and the ViiA7 detection system (Life Technologies). The following primers for RT-PCR were synthesized by Eurofins MWG Operon: col1a1 spanning exons 1 and 2 (forward primer: 5′-TGTTCAGCTTTGTGGACCTC-3′; reverse primer: 5′-TCAAGCATACCTCGGGTTTC-3′), reference gene b-actin (forward primer: 5′-ACCCGCGAGCACAGCTTCTTTG-3′; reverse primer: 5′-ACATGCCGGAGCCGTTGTCGAC-3′), IL-3 (forward primer: 5′-CCTGCCTACATCTGCGAATGAC-3′; reverse primer: 5′-GGGCCATGAGGAACATTCAGAC-3′) reference gene GAPDH (forward primer: 5′-GTCGTGGATCTGACGTGCC-3′; reverse primer: 5′-GATGCCTGCTTCACCACCTT-3′). Fold changes in the relative gene expression levels were determined using the 2-^Δcycle^ ^threshold^ method.

### TCR clonotype analysis.

Total RNA from FACS-sorted CD4^+^ T cell populations was used for 5′ RACE PCR of the TCR-β chain. For cDNA generation, we introduced a template-switching oligonucleotide (TSO; Bio-GGGCUCGGAGAUGUGUAUAAGAGACAGUNNNNUNNNNUNNNNUrGrGrG) that included a unique molecular identifier (UMI). Following reverse transcription, excess TSO was digested using Uracil-DNA Glycosylase (UDG) (New England Biolabs). PCR was performed using the Advantage 2 PCR Kit (Takara) for 20 cycles along with primers specific for the constant region of the Trbc locus (GAAGCCCCTGGCCAAGCACACGAG) as well as a universal forward primer specific for the TSO sequence (GTCTCGTGGGCTCGGAGATGTGTATAAGAGACAGT). The PCR products were purified with AMPure XP beads (Beckman Coulter), and reactions were split into technical replicates for the successive extension PCR (15 cycles) using the Advantage 2 PCR Kit (Takara) and TSO (GTCTCGTGGGCTCGGAGATGTG) and another Tcrb-specific primer (TCGTCGGCAGCGTCAGATGTGTATAAGAGACAGTTGATGGCTCAAACAAGGAG). Barcoding was performed in a third PCR using IDT for Illumina DNA/RNA UD Indexes (10 cycles). Libraries were purified using AMPure XP beads (Beckman Coulter), concentrations were measured with the Qubit dsDNA HS Assay Kit (Thermo Fisher Scientific), and fragment size profiles were assessed using the High Sensitivity D1000 or D5000 ScreenTape Assay (Agilent). Sequencing (paired-end 300 bp) was performed on the Illumina NextSeq 2000 system. UMI-barcoded and paired TCR-sequencing reads were processed using MIXCR (v4.7.0). Mapped TCR-seq data were further analyzed using the immunarch package (v.0.9.1) in R (v4.4.2) to generate clonotype count tables and calculate repertoire diversities. Chao1 diversities were calculated using the repDiversity function of immunarch with downsampled data (with bootstrapping, 1000 iterations) and method “chao1” and plotted using the ggplot2 package (v3.5.2). The circos plot presenting clonotype frequency overlaps were generated using the circlize R package (0.4.16).

### Histological analysis.

One-quarter of each UUO kidney was embedded in paraffin or Tissue-Tek Compound (Sakura Finetek Germany GmbH). Paraffin sections (3 μm) were stained with Masson’s trichrome according to the manufacturer’s protocol (Sigma). Cryosections (3 μm) were used for immunofluorescence staining. After fixation with ice-cold acetone, sections were blocked with super block blocking buffer (Thermo Fischer Scientific) and incubated with an antibody against collagen type 1 (ab21286; Abcam). As detection antibody, Alexa Fluor 594–labeled F(ab’)2 fragments of goat anti-rabbit IgG (A-11072; Invitrogen) were used. The slides were covered with Fluoromount-G Mounting Medium (ThermoFisher) containing DAPI to label DNA. Images were taken with an Axio-Observer-Z1 microscope (Carl Zeiss) for immunofluorescence staining or the Echo Rebel microscope for Masson’s trichrome staining. Signals were semiautomatically analyzed with MetaMorph software (version 4.6; Universal ImagingCorp.). All histological sections were analyzed in a blinded manner.

### Study population and sampling.

Peripheral venous blood was drawn from 37 adult patients with SSc and 37 adult healthy volunteers enrolled at the Asklepios Clinic Bad Abbach from June to October 2024. Patients were stratified according to disease duration and organ involvement.

### Preparation and culture of human PBMCs.

PBMCs were prepared by Ficoll-Paque density gradient centrifugation from heparin-anticoagulated fresh blood samples and cryopreserved. For cryopreservation, PBMCs were resuspended in FCS with 10% DMSO at a concentration of 1 × 10^6^ to 2 × 10^6^ cells/mL, frozen at −80°C for 2 days, and then transferred into liquid nitrogen. The cells were thawed in a 37°C water bath and washed 3 times with medium. The viability was controlled by trypan blue staining and was 90%–95%. PBMCs (500,000/well) were cultured in 300 μL medium/well (RPMI-1640 with 10% FCS, 1% penicillin/streptomycin and 1% L-glutamine; all Gibco) with or without 5 μg/mL anti-CD3 (clone OKT3, eBiosciences) at 37°C for 24 hours. Thereafter, supernatants were recovered and analyzed by ELISA.

### ELISA for detection of human IL-3.

For measurement of IL-3, ELISA plates (NUNC Maxisorb, Thermo Fisher Scientific) were coated with a capture anti–IL-3 antibody (clone P8C11; 5 μg/mL in PBS) in 100 μL/well at room temperature overnight. Plates were washed 3 times with PBS/0.05% Tween-20, blocked with PBS containing 1% BSA at room temperature for 1 hour, and then washed again with PBS. Samples were preincubated with 100 μg/mL mouse IgG1, κ isotype control antibody (MOPC21, BioXCell) at room temperature for 1 hour and added to the plates at room temperature for 2 hours (100 μL/well, diluted in PBS containing 1% BSA). Recombinant human IL-3 (BioLegend) was diluted from 7.8 to 500 pg/mL in PBS/1% BSA and served as standard. After washing, plates were incubated with 400 ng/mL HRP-labeled detection anti–IL-3 antibody (clone 13, InVivo BioTech Services GmbH, Germany) (100 μL/well) at room temperature for 1.5 hours, and color reaction was performed with TMB Substrate Solution (BioLegend) according to the manufacturer’s protocol.

### Statistics.

All data are shown as the mean ± SEM. Statistical significance was defined at a *P* value of 0.05 or less. Statistical analysis was performed with GraphPad Prism 9.3.1. Statistical tests are provided in each figure legend and include 1-way ANOVA with multiple comparisons, unpaired 2-sided t test, and Welch’s 2-sided *t* test.

### Study approval.

All animal studies were approved by the “Regierung der Oberpfalz” (Würzburg, Germany; Az. 2532-2-1281) and performed in accordance with institutional guidelines.

The human study was approved by the Research Ethics Committee of the University Hospital Regensburg and performed in accordance with all relevant ethical regulations for work with humans. Written informed consent and consent to publish were obtained from all enrolled patients and healthy volunteers.

### Data availability.

The authors declare that all data supporting the findings of this study are available in the main text or the supplemental material, including the Supporting Data Values file. Raw data from RNA-seq have been deposited in the NCBI’s Gene Expression Omnibus (GEO) database with accession GSE312018.

## Author contributions

MM conceived and supervised the study. SB, AF, SN, KS, YT, FWK, SB, KL, CB, MR, FVS, MB, KR, and MM performed and analyzed experiments. FG and MF provided patient samples and clinical data. SB and MM drafted the manuscript with input from all authors.

## Funding support

- Deutsche Forschungsgemeinschaft (German Research Foundation) – Project-ID 509149993, TRR 374.

## Supplementary Material

Supplemental data

Supporting data values

## Figures and Tables

**Table 1:**
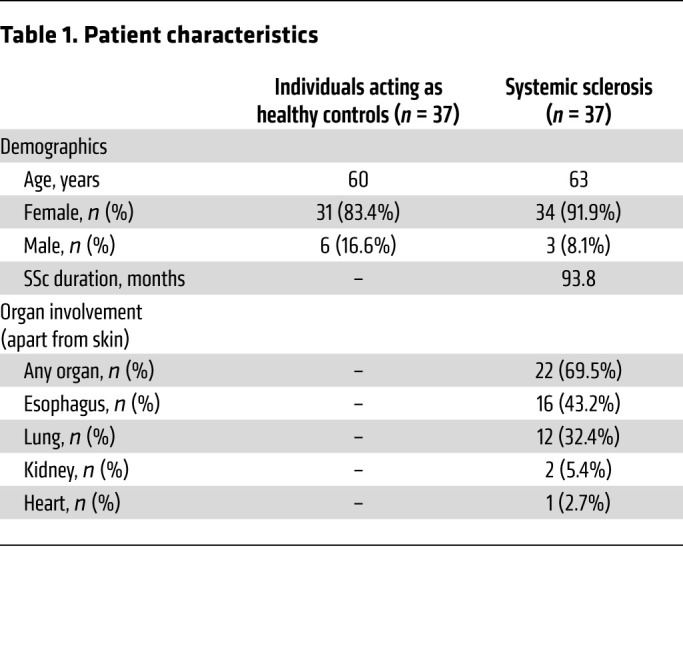
Patient characteristics

## Version 1 03/16/2026

Electronic publication
