# Supplementary material for: Fibrosis-memory is mediated by IL-3–producing T cells and drives progression of fibrosis
Source: J Clin Invest. 2026 Mar 16;136(6):e192095. doi: 10.1172/JCI192095 (PMC12987615; doi:10.1172/JCI192095)
Supplement: Supplemental data [file jci-136-192095-s240.pdf]

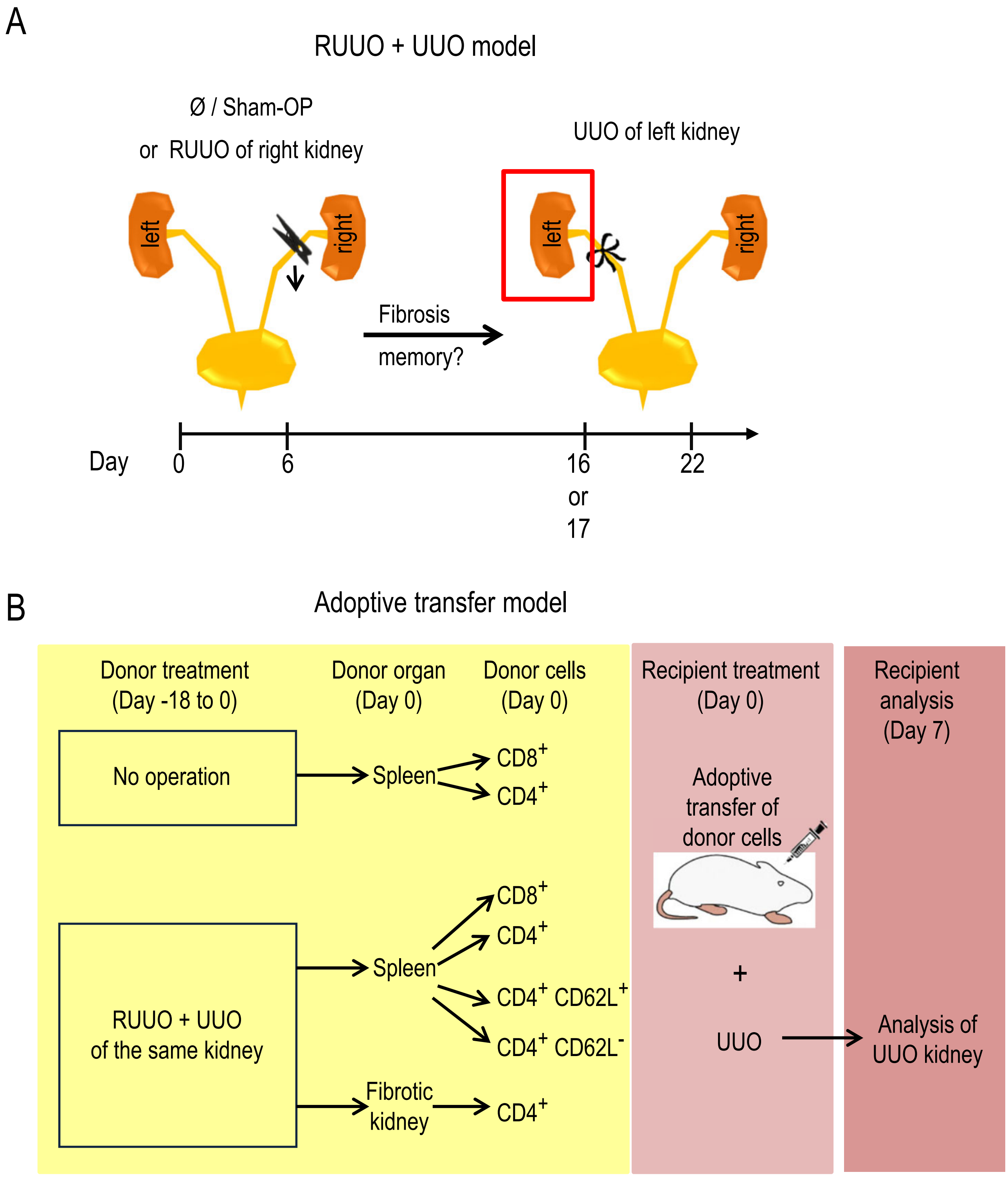


Supplemental Figure 1

**Supplemental Figure 1**

**Schematic overview of experimental setups.**

(A) Fibrosis-memory model with two consecutive renal injuries (RUUO+UUO model) used in Fig. 1A, E; Fig. 2; Fig. 6A, B. Clamp placement on the right ureter at day 0 with clamp relocation on days 2 and 4 to avoid strictures. Clamp removal at day 6. Ligation of the left ureter at day 16 or 17 and analysis of the left UUO-kidney at day 22.

(B) Adoptive transfer of T cells used in Fig. 3; Fig. 4I, Fig. 5 and Fig. 6C, D. Donor mice remained naïve (no operation) or suffered from a renal fibrosis induced by a reversible UUO (RUUO) of the right kidney from day -18 to day-12 followed by an UUO (UUO) of the same kidney from day -7 to 0. Various T cell subsets were purified from the spleen or fibrotic right kidney of donor mice on day 0. The purified T cells were adoptively transferred to recipients by intravenous injection on day 0 and UUO was induced in the recipients on day 0. Analysis of the recipient's UUO-kidney at day 7.

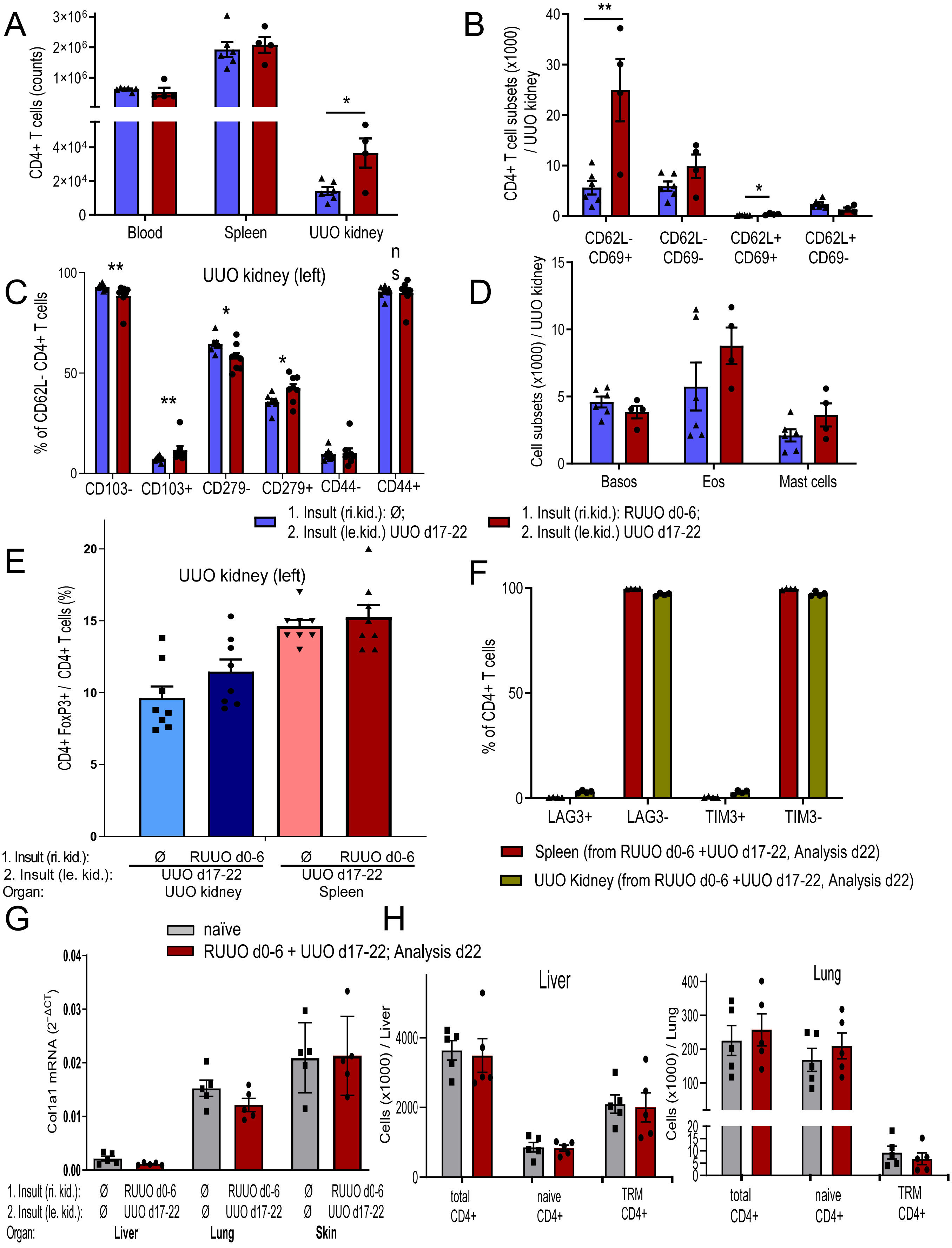


Supplemental Figure 2

**Supplemental Figure 2**

**Analysis of mice with fibrosis-memory**

Mice underwent RUUO of the right kidney from day 0-6 (first insult) followed by UUO of the left kidney from day 17-22 (second insult) (n=6). Control mice only underwent UUO of the left kidney from day 17-22 (n=4). (A) Absolute $CD4^+$ T cell counts in blood, spleen and left UUO-kidney at day 22. (B-D) Subsets of $CD4^+$ T cells (B), subsets of $CD62L^-CD4^+$ T cells (C), Basophils (Basos), eosinophils (Eos), and mast cells (D) in the left UUO-kidney at day 22. (E) Quantification of Treg cells in the left UUO-kidney at day 22 (n=8). (F) Percentage of $CD4^+$ T cells expressing TIM-3 and LAG-3 in spleen and UUO-kidney (n=4). (G) Quantification of collagen-1a1 mRNA expression in the liver, lung and skin of naïve mice (Ø) or mice with two fibrotic insults to the kidneys (n=5). (H) Total, naïve ($CD62L^+$) and tissue resident memory ($CD69^+$ $CD62L^-$) (TRM) in the liver and lung of naïve mice and mice with two fibrotic insults to the kidneys (n=5). Data are represented as mean ± SEM. One-way ANOVA. *P<0.05; **P<0.01.

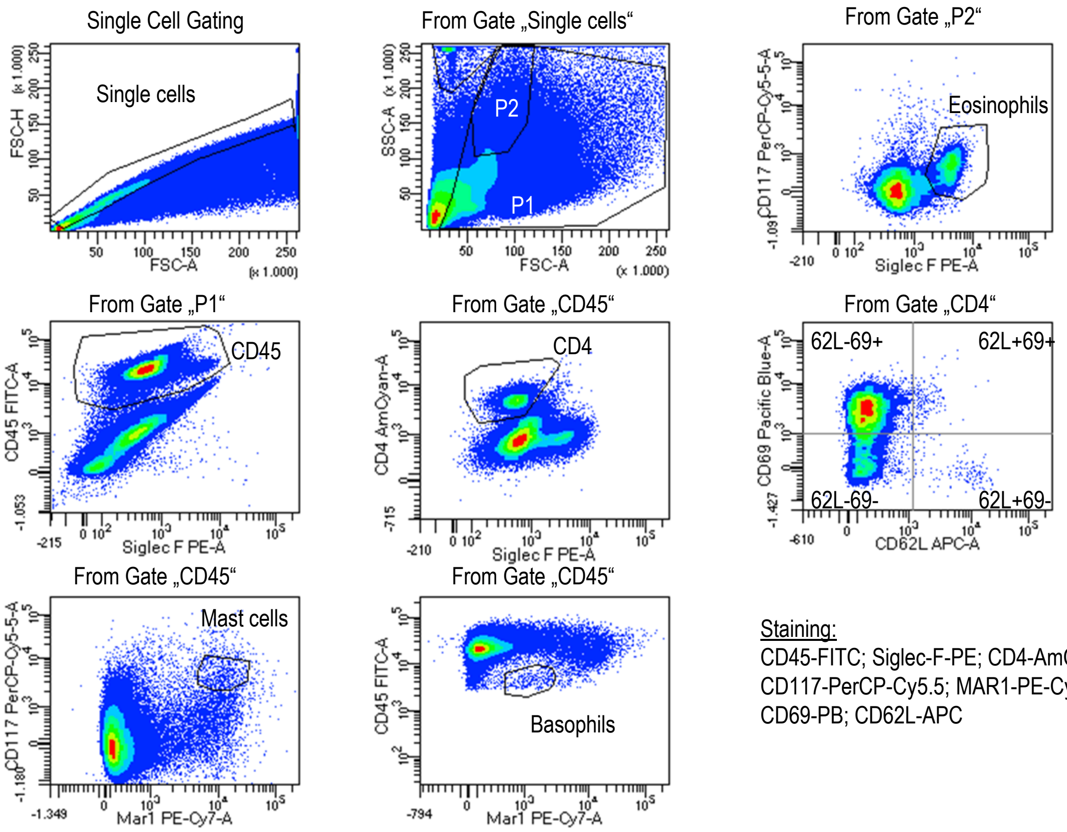


Staining:
CD45-FITC; Siglec-F-PE; CD4-AmCyan; CD117-PerCP-Cy5.5; MAR1-PE-Cy7; CD69-PB; CD62L-APC

Supplemental Figure 3
Gating strategy and antibody panel used for flow cytometry.

Supplemental Figure 3

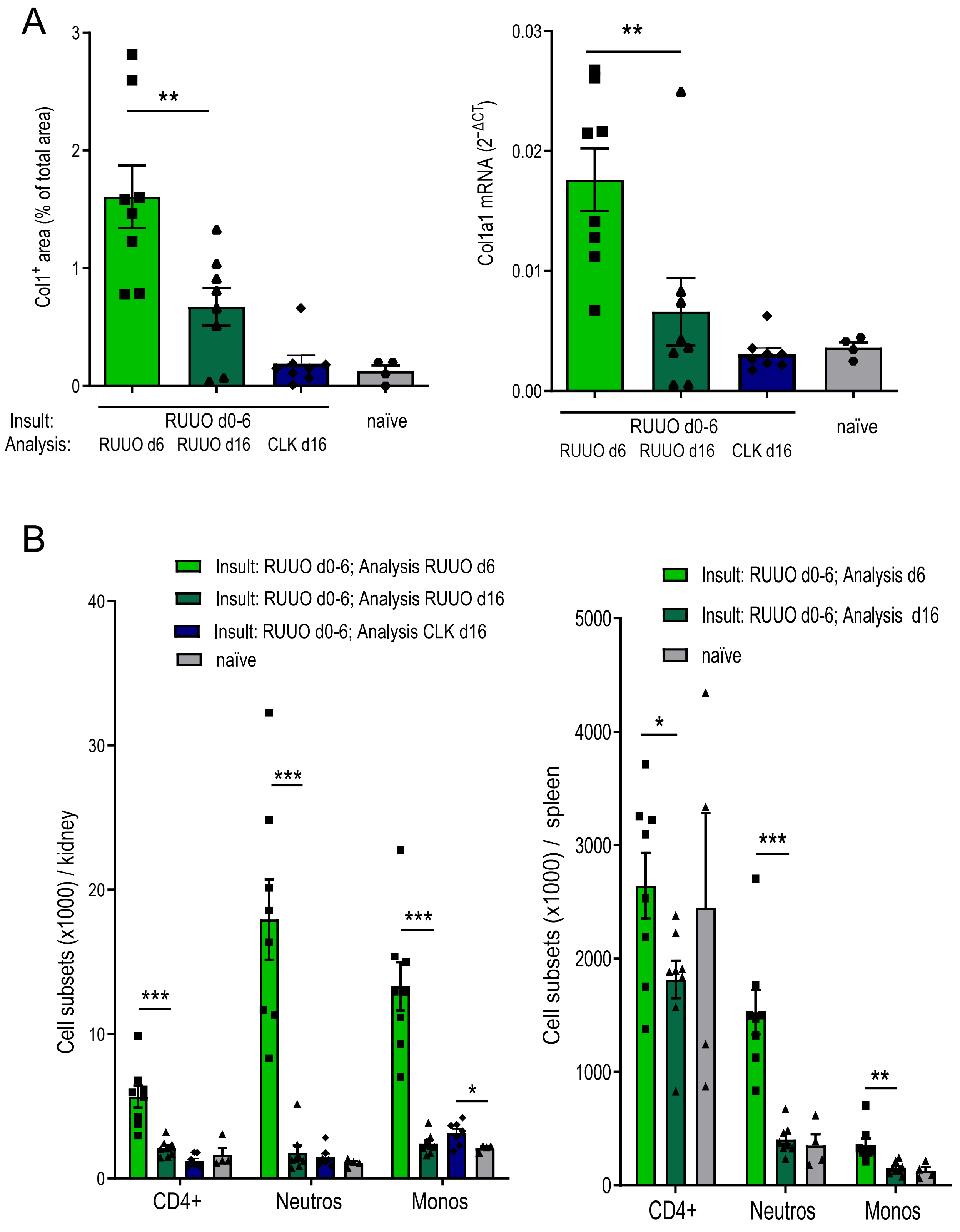


Supplemental Figure 4

**Supplemental Figure 4**

**Characterization of mice with RUUO from day 0-6.**

RUUO was performed from day 0-6 (n=8 / group). RUUO-kidneys and spleens were analyzed on day 6 and on day 16. In addition, contralateral kidneys (CLK) were analyzed on day 16. Kidneys and spleens from non-operated naive mice (n=4) served as control. (A) Quantification of collagen-1 (Col1) and col1a1 mRNA expression in the kidneys. (B) Quantification of $CD4^+$ T cells, neutrophils (Neutros) and monocytes (Monos) in the kidneys and spleen. Data are represented as mean ± SEM. One-way ANOVA between RUUO d6 and d16 and between CLK and naïve kidney. Data are represented as mean ± SEM. One-way ANOVA with multiple comparisons for spleen. *P<0.05; **P<0.01; ***P<0.001.

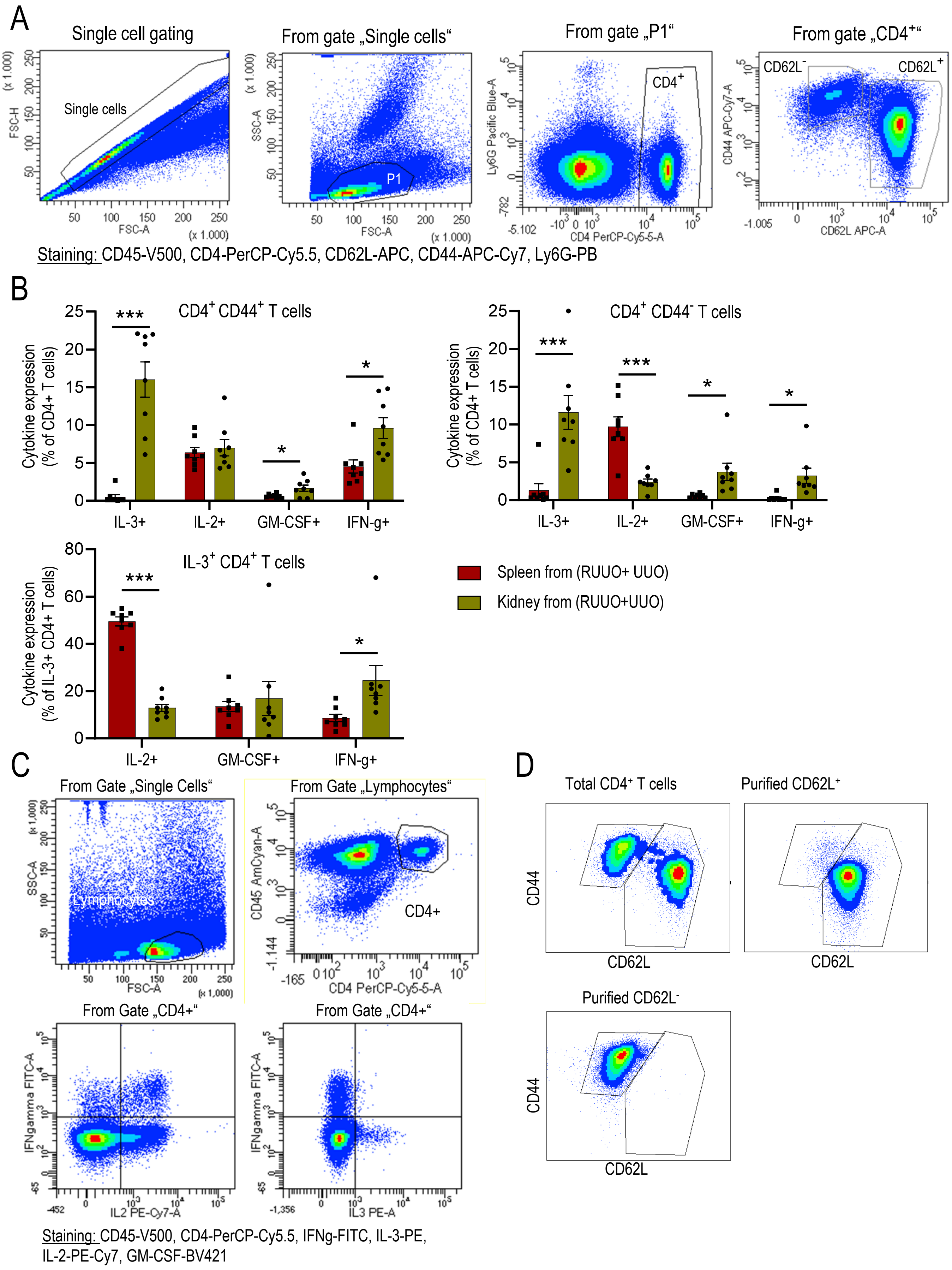


Supplemental Figure 5

**Supplemental Figure 5**

**Gating strategies, immunophenotyping and purity of sorted $CD4^+$ T cells.**

(A) Gating strategy and antibody panel used to identify naïve ($CD62L^+$) and memory ($CD62L^-$) $CD4^+$ T cells in the spleen and kidney. (B) Co-expression of IL-2, GM-CSF and IFN-gamma by IL-$3^+$ $CD4^+$ T cells in the spleen and kidney of mice with fibrosis-memory (for details see Fig. 4B). Gating strategy and antibody panel used to identify cytokine-expressing $CD4^+$ T cells. (C) Purity of naïve ($CD62L^+$) and memory ($CD62L^-$) $CD4^+$ T cells for adoptive transfer experiments. Data are represented as mean ± SEM. One-way ANOVA. *$P<0.05$; ***$P<0.001$.

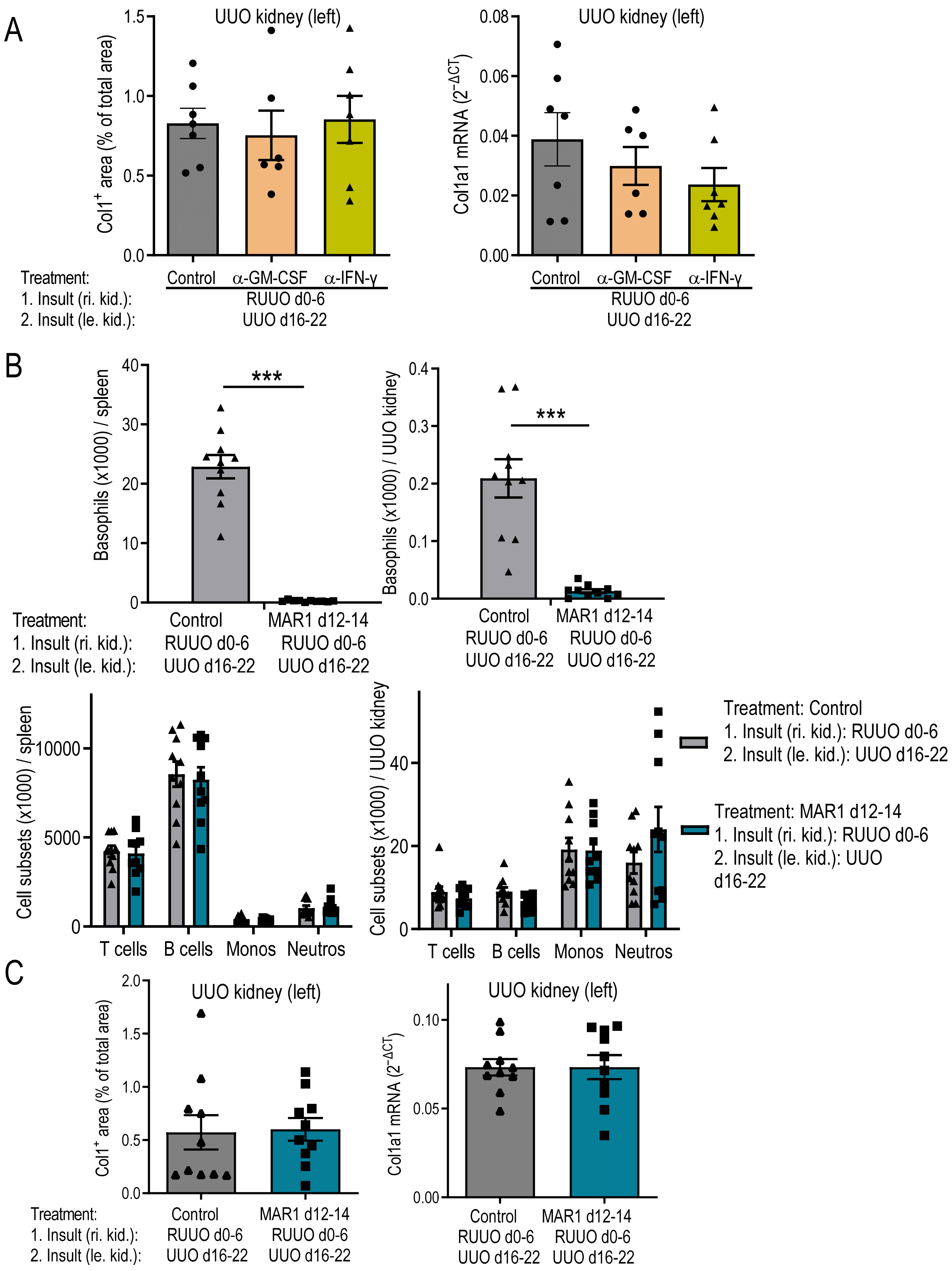


Supplemental Figure 6

**Supplemental Figure 6**

**Blockade of cytokines and depletion of basophils in mice with fibrosis-memory.**

RUUO of the right kidney was performed from day 0-6 and UUO of the left kidney from day 16-22. (A) On days 15, 17, 19 and 21, mice were injected with blocking antibodies against GM-CSF or IFN-gamma or received an isotype control antibody (n=6 per group). Quantification of collagen-1 (Col1) and col1a1 mRNA expression in the left UUO-kidneys on day 22. (B-C) On days 12-14 mice were injected with the basophil-depleting antibody MAR-1 (n=10) or received an isotype control antibody (Control; n=10). (B) Quantification of basophils, T cells, B cells, monocytes (Monos) and neutrophils (Neutros) in the spleen and UUO-kidney on day 22. (C) Quantification of collagen-1 (Col1) and col1a1 mRNA expression in the left UUO-kidneys on day 22. Data are represented as mean ± SEM. Unpaired one-sided t-test. ***P<0.001.

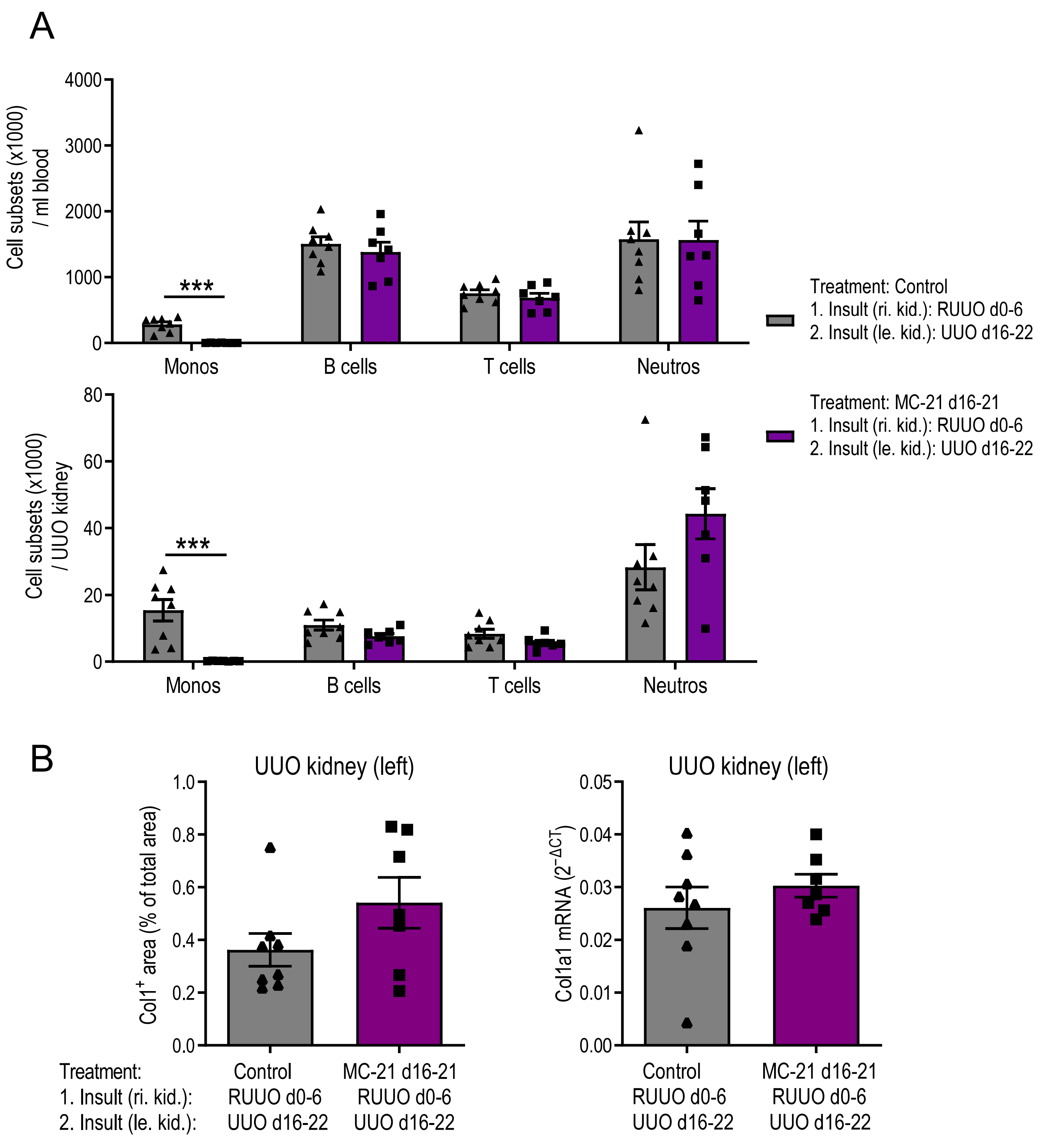


Supplemental Figure 7

**Supplemental Figure 7**

**Depletion of $CCR2^+$ monocytes in mice with fibrosis-memory.**

RUUO of the right kidney was performed from day 0-6 and UUO of the left kidney from day 16-22. On days 16-21 mice were injected with the monocyte-depleting CCR2 antibody MC-21 (n=7) or received an isotype control antibody (Control; n=8). (A) Quantification of $Ly6C^+$ monocytes (Monos), T cells, B cells and neutrophils (Neutros) in the peripheral blood and UUO-kidney on day 22. (B) Quantification of collagen-1 (Col1) and col1a1 mRNA expression in the left UUO-kidneys on day 22. Data are represented as mean ± SEM. Unpaired one-sided t-test. ***P<0.001.

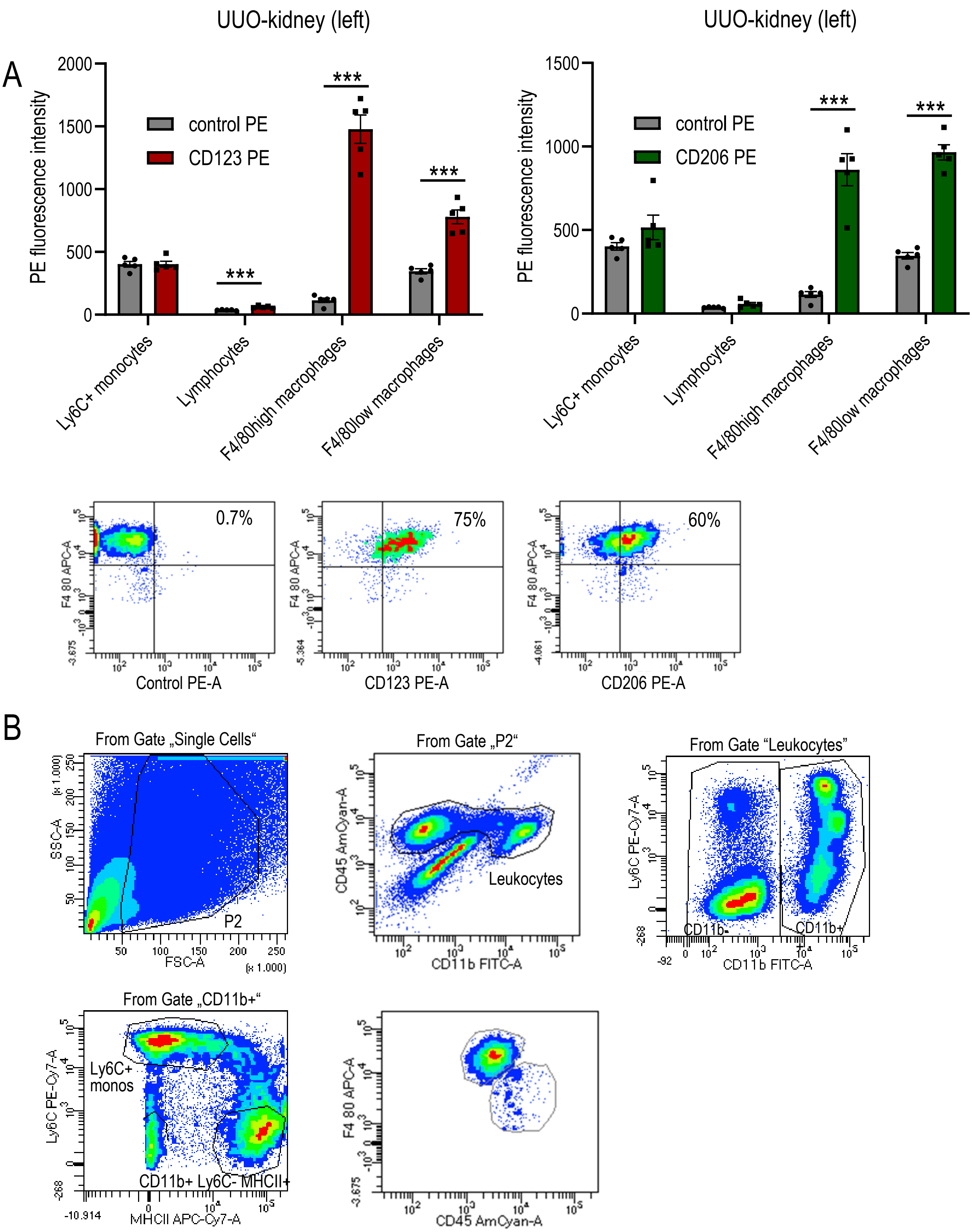


Staining: CD11b-FITC; CD123-PE; F4/80-APC, MHC-II-APC-Cy7; Ly6C-PE-Cy7; CD45-AmCyan

# Supplemental Figure 8

**Supplemental Figure 8**

**F4/80-high macrophages strongly express IL-3R.**

(A) UUO was performed on day 0 (n=3) and leukocyte subsets were analyzed in the UUO-kidney by flow cytometry on day 4. Expression of CD123 (IL-3-receptor alpha chain) and CD206 was determined on various leukocyte subsets (shown as mean fluorescence intensity). Representative dot plots showing expression of CD123 and CD206 (right panel). (B) Gating strategy and antibody panel used for flow cytometry. Data are represented as mean ± SEM. Unpaired two-sided t-test. ***P<0.001.
